# Phenotypic and functional alteration of CD45+ immune cells in the decidua of preeclampsia patients analyzed by mass cytometry (CyTOF)

**DOI:** 10.3389/fimmu.2022.1047986

**Published:** 2023-01-06

**Authors:** Min Fu, Xiaowei Zhang, Chunfeng Liu, Jinli Lyu, Xinyang Liu, Shilin Zhong, Yiheng Liang, Ping Liu, Liting Huang, Zhansong Xiao, Xinxin Wang, Xiaoling Liang, Hao Wang, Shangrong Fan

**Affiliations:** ^1^ Department of Obstetrics and Gynecology, Peking University Shenzhen Hospital, Shenzhen, Guangdong, China; ^2^ Department of Reproductive Medicine, Peking University Shenzhen Hospital, Shenzhen, Guangdong, China; ^3^ Institute of Obstetrics and Gynecology, Shenzhen Peking University - Hong Kong University of Science and Technology (PKU-HKUST) Medical Center, Shenzhen, Guangdong, China; ^4^ Shenzhen Key Laboratory on Technology for Early Diagnosis of Major Gynecological Diseases, Shenzhen, Guangdong, China; ^5^ Department of Obstetrics and Gynecology, Peking University Shenzhen Hospital, Clinical College of Anhui Medical University, Shenzhen, Guangdong, China; ^6^ The Assisted Reproduction Center, Northwest Women’s and Children’s Hospital, Xi’an, China; ^7^ Department of Obstetrics and Gynecology, Sun Yat‐Sen Memorial Hospital, Guangzhou, China

**Keywords:** preeclampsia, cytometry by time-of-flight mass spectrometry (CyTOF), Breg-like cell, CD11c+CD8+T cell, NK cells, functional marker

## Abstract

Preeclampsia (PE) is a severe placenta-related pregnancy disease that has been associated with maternal systemic inflammation and immune system disorders. However, the distribution and functional changes in immune cells of the maternal–placental interface have not been well characterized. Herein, cytometry by time-of-flight mass spectrometry (CyTOF) was used to investigate the immune atlas at the decidua, which was obtained from four PE patients and four healthy controls. Six superclusters were identified, namely, T cells, B cells, natural killer (NK) cells, monocytes, granulocytes, and others. B cells were significantly decreased in the PE group, among which the reduction in CD27+CD38+ regulatory B cell (Breg)-like cells may stimulate immune activation in PE. The significantly increased migration of B cells could be linked to the significantly overexpressed chemokine C-X-C receptor 5 (CXCR5) in the PE group, which may result in the production of excessive autoantibodies and the pathogenesis of PE. A subset of T cells, CD11c+CD8+ T cells, was significantly decreased in PE and might lead to sustained immune activation in PE patients. NK cells were ultimately separated into four subsets. The significant reduction in a novel subset of NK cells (CD56-CD49a-CD38+) in PE might have led to the failure to suppress inflammation at the maternal–fetal interface during PE progression. Moreover, the expression levels of functional markers were significantly altered in the PE group, which also inferred that shifts in the decidual immune state contributed to the development of PE and might serve as potential treatment targets. This is a worthy attempt to elaborate the differences in the phenotype and function of CD45+ immune cells in the decidua between PE and healthy pregnancies by CyTOF, which contributes to understand the pathogenesis of PE, and the altered cell subsets and markers may inspire the immune modulatory therapy for PE.

## 1 Introduction

Preeclampsia (PE) is a multiorgan syndrome that affects 2%–8% of pregnancies worldwide (1), and it is the second leading cause of maternal death globally (2). PE is diagnosed by new-onset hypertension developing on or after 20 weeks of gestation and new-onset proteinuria or, in its absence, thrombocytopenia, impaired liver function, renal insufficiency, pulmonary edema, or cerebral or visual disturbances (2). In PE patients, the physiological changes in uterine spiral arteries are restricted to the decidua, whereas in normal pregnancy, they extend proximally into the myometrium. This superficial invasion of the decidua results in narrow and undilated proximal segments of the spiral arteries, which ultimately leads to uterine hypoperfusion and further results in fetal growth restriction or adverse pregnancy outcomes. Short-term and long-term follow-up research on women with PE suggested that these women had apparent clinical or subclinical symptoms of metabolic syndrome (3). Although the etiology of PE remains elusive, a dysregulated maternal immune system and systemic inflammation have been recognized as crucial contributors to the pathophysiological programming of PE (4).

Inflammatory and immune cells play important roles in embryo implantation, placental development, and delivery. Immune tolerance is in dynamic equilibrium during normal pregnancy, whose imbalance will lead to an adverse pregnancy. In early pregnancy, decidual immune cells, as indispensable parts of the maternal–fetal interface, account for 30%–40% of the total decidual cells, among which decidual natural killer (dNK) cells comprise the majority (~70%) of immune cells, followed by decidual macrophages (20%–25%) and T cells (3%–10%) (5). These leukocytes are present in the decidua throughout pregnancy, although the population frequencies change. For instance, dNK cells and decidual macrophages are more abundant at earlier stages of pregnancy than at term (6). In the first trimester, dNK cells are enriched around spiral arteries near the implantation site and secrete chemokines and angiogenic factors involved in trophoblast infiltration and angiogenesis, such as vascular endothelial growth factor (VEGF), placental growth factor (PlGF), and angiotensin II (AngII) (7). Similar to dNK cells, decidual macrophages aid in remodeling spiral arteries and trophoblast invasion. They phagocytose apoptotic trophoblasts to prevent the activation of pro-inflammatory pathways in the decidua and produce indoleamine 2,3-dioxygenase (IDO) to suppress T-cell activation (8). Moreover, imbalances in the local and peripheral T Helper (TH)1/TH2/regulatory T cell (Treg)/TH17 ratio are reported to be associated with several pregnancy complications, such as unexplained recurrent pregnancy loss, PE, and preterm birth (9). Accumulating evidence supports the idea that B cells play seemingly conflicting roles during pregnancy, either protecting or harming the fetus. However, research on the distribution of B cells and their participation in immune tolerance induction toward the fetus is emerging (10).

The innate and adaptive immune systems heavily contribute to PE, with marked shifts related to bone marrow, T cells, B cells, granulocytes, and neutrophils on the maternal–placental interface (5, 11, 12). The functions of transmembrane proteins (13), mucins (14), and chemokines (15) in immune cells from PE patients have been explained in recent years. Under the limitation of the gating strategy for flow cytometry, most of these studies can only focus on one or two types of immune cells, such as T cells (16, 17), NK cells (18), and macrophages (19). Advanced techniques, such as single-cell RNA sequencing (scRNA-seq), have been generated and applied to investigate the phenotype and function of cells in specific tissues, but they cannot explain the protein expression, which can be investigated by flow cytometry. Although a systematic analysis of peripheral blood immune cells from PE patients has been reported (20), it cannot reflect the immune response of the maternal–fetal interface, which is important to the pathogenesis of PE. To date, there have been few in-depth and systematic studies on the immune cells of the decidua in PE, which is very important for the mechanistic study of PE.

Cytometry by time-of-flight mass spectrometry (CyTOF) is an advanced high-throughput mass spectrometry technique that uses monoclonal antibodies (mAbs) tagged with heavy atoms (lanthanides, metals, and actinides) to replace traditional fluorescently labeled antibody markers and combines with inductively coupled plasma time-of-flight mass spectrometry (ICP-TOF-MS) to achieve the rapid detection of multidimensional biomarkers in a single cell. Therefore, it not only continues the characteristics of traditional flow cytometry but also has the high-resolution ability of mass spectrometry detection, which can realize single-cell detection of multichannel protein expression profiles (21). The ability of CyTOF to detect multiple parameters of single cells makes it possible to finely distinguish and group the cell phenotypes. Hence, it has been widely used in many fields, such as immunology, tumor, blood disease, nerve, infection, and drug pharmacology research. Herein, we harnessed the power of CyTOF and tried to reveal the immune phenotypic characteristics of the decidua in PE.

## 2 Materials and methods

### 2.1 Recruitment of participants

PE patients (n = 4) and healthy pregnancies (n = 4) were recruited by the Department of Obstetrics and Gynecology, Peking University Shenzhen Hospital, Shenzhen, China, from December 2020 to August 2021. The range of delivery in gestation weeks was 36~39. PE was diagnosed with blood pressure ≥140/90 mmHg after 20 weeks in the absence of preexisting nephropathy-associated proteinuria or essential hypertension. Pregnancies that were normotensive throughout gestation were selected as healthy controls. All of the PE patients and controls had no adverse pregnancy history, such as recurrent spontaneous abortion, stillbirth, or neonatal death and no autoimmune diseases and delivered at term. All of the collection protocols were approved by the Research Ethics Committee of Peking University Shenzhen Hospital. Written informed consent was obtained from all patients.

### 2.2 Sample collection and processing

Decidual tissues of each participant were collected during surgery. Samples were washed with sterile saline and collected in ice-cold MACS Tissue storage solution (catalog no. 130-100-008, Miltenyi, Germany). Then, they were well sealed and sent to a 4°C refrigerator within 30 min, and transported to the laboratory for processing within 48 h using ice (2-8°C). A single-cell suspension was prepared as follows. Decidual tissues were washed twice using RPMI 1640 medium, transferred to Biotenyi Biotec C tube after cutting into pieces, added with C1 enzyme (0.5 mg/ml) and DNase (20 µg/ml), and digested at 37°C with 145 rpm for 1 h. The digested solution was filtered through a 70-µm sieve and washed with RPMI 1640 medium. Subsequently, 1 ml Ammonium-Chloride-Potassium red blood cell (ACK RBC) lysate was added and mixed well. The cracking was stopped by adding Fluorescence Activated Cell Sorting (FACS) buffer after 1–2 min of being stationary. The solution was centrifuged at 400 × g for 5 min, and the supernatant was discarded. The cell precipitates were resuspended by adding 4 ml FACS buffer, and the cells were counted. As a result, 3 × 10^6^ cells were collected for subsequent staining.

### 2.3 CyTOF staining and data acquisition

Based on the cell types we were interested in in decidual tissue reported in previous studies, we selected 42 metal-conjugated antibodies as markers to identify the cells in this study. Detailed information on the 42 markers is listed in **Table S1**. Prior to the formal experiments, the optimal dilutions of the antibodies were determined. First, antibodies were conjugated to metal tags using the Maxpar Antibody Labeling Kit (Fluidigm, South San Francisco, CA, USA). The processed antibodies were stored in phosphate-buffered saline (PBS)-based antibody stabilization solution (Candor Biosciences, Constance, Germany) supplemented with 0.05% NaN_3_ at 4°C. The concentration of mass-tagged antibodies was then assessed by NanoDrop and adjusted to 200 mg/ml antibody stabilization solution. Different diluted concentrations of conjugated antibodies (1:50, 1:100, 1:200, 1:400, 1:800) were tested on peripheral blood samples or standard cell lines using CyTOF. The output results of all antibodies for formal experiments are presented in **Figure S1**. The optimal dilutions were determined by clear clustering according to the antibody manuals.

The obtained single cells were subjected to live/dead staining using 100 μl of 0.25 μM Cell-ID™ Cisplatin-194Pt (Fluidigm, South San Francisco, CA, USA) for 5 min on ice and washed with FACS buffer before being blocked on ice for 20 min. Then, the cell surfaces were stained with 50 μl antibody mix on ice for 30 min and washed twice with FACS buffer. Maxpar^®^ Fix and Perm Buffer containing 250 nM Cell-ID™ Intercalator-Ir (Fluidigm, South San Francisco, CA, USA) was subsequently added and incubated at 4°C overnight to fix the cells and stain DNA. Then, 100 μl of intracellular antibody-staining cocktail was added to each sample for resuspension and incubated on ice for 30 min. After multiple washes with FACS buffer and ultrapure H_2_O, the cells were resuspended in 1 ml Milli-Q water in each sample, 20% EQ Four Element Calibration Beads (Fluidigm) were added, and the samples were filtered with a 35-μm nylon mesh filter cap (Falcon, BD Bioscience). Finally, the prepared samples were sent for CyTOF mass cytometer analysis. As a loading control, a 1:10 dilution of EQ Four Element Calibration Beads (Fluidigm) was used. All data were produced on a Helio3 CyTOF Mass Cytometer (Fluidigm).

### 2.4 Bioinformatic analysis of mass cytometry data

CyTOF analyses were performed by PLTTech Inc. (Hangzhou, China) according to a previously described protocol (22–24). The original data of each marker were first normalized by a bead-based normalizer and then debarcoded. Manual gating was performed next by FlowJo V10 software using different channels, such as DNA191 vs. DNA193 for cell debris removal, 194Pt for dead cell removal, and adhesion cell removal. As a result, effective target single cells were obtained. A total of 100,000 effective cells were randomly selected from each sample, and the selected cells of all samples were mixed together for clustering. All classification and function markers were applied for clustering and visualization after arcsinh transformation (with the cofactor set to 5). The PhenoGraph (25) algorithm was used to cluster cells. A total of 30,000 cells from each group were randomly selected for visualization by t-distributed stochastic neighbor embedding (t-SNE) using the R package cytofkit and set Kmeans = 30 and R = 1 (26). Cells were annotated with classic markers. Subtype cells were defined by specific markers. For the cell types we were interested in for further analysis, FlowJo V10 software was used to gate the target cells, and then Kmeans = 30 and R = 1 were used to perform the recluster. The median value of each marker in each cell type and subtype was used to determine the correction (27) by hierarchical clustering.

### 2.5 Statistical analysis

Unpaired-sample Student’s t-test or Mann-Whitney U test was performed to compare the differences between groups as fit. Statistical analyses were conducted using R 4.0.0 (R Core Team, Vienna, Austria). All statistical tests were two-sided, and p < 0.050 was considered to be statistically significant.

## 3 Results

### 3.1 Phenotypic and functional markers of CD45+ decidual immune cells are altered in PE

**Figure 1:**
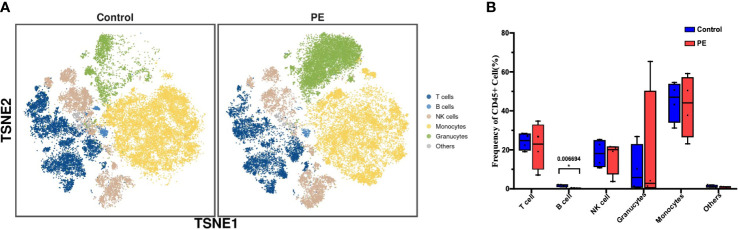
The phenotype and functional markers of CD45+ decidual immune cells are altered in PE. **(A)** t-SNE plot of all CD45+ cells from the PE and control groups that merged into six superclusters, namely, T cells, B cells, natural killer (NK) cells, monocytes, granulocytes, and others. **(B)** Bar plot of the frequencies of CD45+ cells in all superclusters. The blue bar and red bar denote the control and PE groups, respectively. * stands for p-value <0.01. The number above the asterisk is the exact p-value.

Decidual immune cells were determined by 42 functional markers in healthy and PE puerpera. Leukocytes that presented CD45+ were captured and defined as immune cells. All of the immune cells were clustered and visualized as a two-dimensional map by t-SNE. In total, 32 distinct clusters were obtained (**Figure S2**) and annotated by the expression of markers and literature evidence (**Table S2**). Subsequently, they were merged into six superclusters, namely, T cells, B cells, NK cells, monocytes, granulocytes, and others (**Figure 1A**). The proportion of all of the superclusters was not significantly different between the PE and control groups, except for B cells, which were significantly decreased in the PE group compared to the control group (p < 0.05) (**Figure 1B**). Differences in the expression of functional markers between the two groups were also detected. For all CD45+ immune cells, the expression of Tim-3, CD33, and C-X-C receptor 5 (CXCR5) was significantly higher in PE (p < 0.05) (**Figure S3**). By examining each supercluster, the expression of tumor necrosis factor receptor superfamily member 4 (OX40), C-C Motif Chemokine Receptor 4 (CCR4), CD11c, CD14, CD25, CD28, CD4, CD68, CD69, cytotoxic T-lymphocyte-associated protein 4 (CTLA-4), CXCR3, forkhead box P3 (FoxP3), gamma delta T-cell receptor (gdTCR), lymphocyte-activation gene 3 (LAG3), and programmed death-ligand 1 (PD-L1) was significantly changed between the two groups in at least one supercluster (p < 0.05) (**Table S3**). The results suggested that although the proportion of the most immune cell superclusters in the PE group did not differ from that in the control group, the immune function was changed.

### 3.2 Total B cells decreased while migrating B cells increased in PE

**Figure 2:**
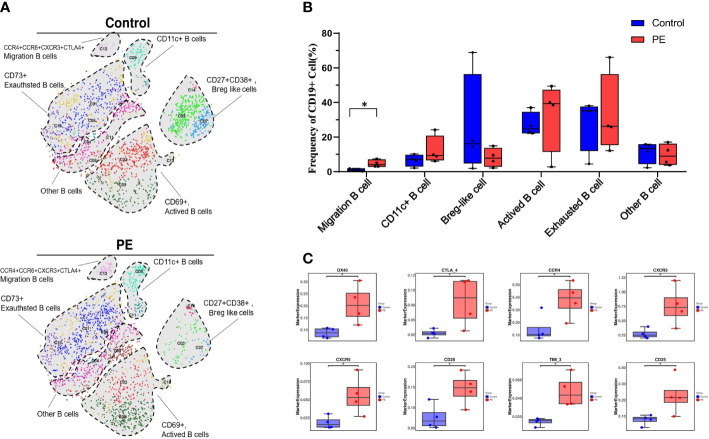
Total B cells decreased while the migration of B cells increased significantly in PE patients compared with healthy controls. **(A)** t-SNE plot of 14 clusters of B cells annotated by CD45+CD19+CD66b-CD3- from the PE and control groups and merged into six subsets, namely, CCR4+CCR5+CXCR3+CTLA-4 migrating B cells, CD11c+ B cells, CD27+CD38+ Breg-like cells, CD69+ activated B cells, CD73+ exhausted B cells, and other B cells. Each color represents a cluster, and each shaded area circled by the dashed line represents a subset. **(B)** The frequencies of B-cell subsets are shown in the bar plot. **(C)** The expression of significantly changed markers in the PE and control groups. The blue bar and red bar in panels B and C denote the control and PE groups, respectively. * stands for p-value <0.01.

As B cells (defined by CD45+CD19+CD66b-CD3-) were the only immune cell supercluster that significantly differed between the PE and control groups, they were reclustered to explore their distinctive characteristics. As a result, 14 clusters were obtained (**Figure S4**). Based on the expression of the specific markers, they were grouped into CCR4+CCR5+CXCR3+CTLA-4 migrating B cells, CD11c+ B cells, CD27+CD38+ regulatory B cell (Breg)-like cells, CD69+ activated B cells, CD73+ exhausted B cells, and other B cells (**Figure 2A**). Among them, the median frequencies of Breg-like and exhausted B cells, annotated as CD27+CD38+ and CD73+, respectively, were decreased in the PE group, but no significant differences were detected. Moreover, the number of migrating B cells annotated by CCR4+CCR6+CXCR3+CTLA-4+ increased significantly in the PE group (p < 0.05). The activated B-cell subsets also increased in PE, but with no significant differences (**Figure 2B**). Although the total B-cell frequency decreased in the PE group, their surface molecule expression showed an overall increasing trend in PE, including the activation markers CD25 and CD28, the costimulatory receptor OX40, the chemokine receptors CXCR3, CCR4, and CXCR5, and the immune checkpoints Tim-3 and CTLA-4. All of the above markers were significantly increased in the PE group (p < 0.05) (**Figure 2C**).

### 3.3 The CD11c+CD8+ T-cell subset was significantly reduced in PE

**Figure 3:**
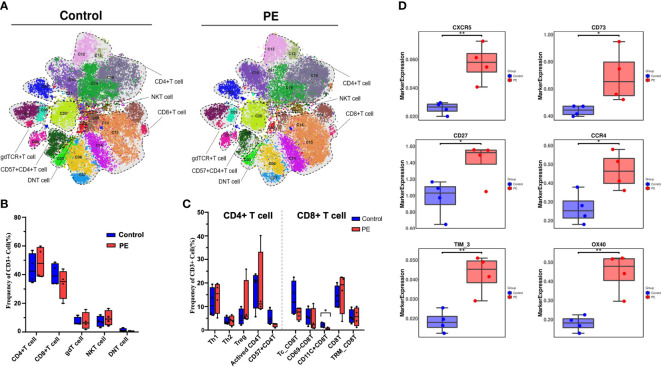
T cells were further selected and reclustered to show the alteration of T-cell subsets in PE patients, and the CD11c+CD8+ T-cell subset was significantly reduced in PE. **(A)** t-SNE plot of 21 clusters of CD3+ T cells from the PE and control groups that were merged into five superclusters, namely, CD4+ T cells, CD8+ T cells, gdT cells, DNT cells, and NKT cells. Each color represents a cluster, and each shaded area circled by the dashed line represents a subset. **(B)** Bar plot of the frequencies of T-cell superclusters is shown. **(C)** Bar plot of the frequencies of the subsets classified as CD8+ T cells and CD4+ T cells is shown. **(D)** The markers expressed in all T cells that differed significantly in the PE and control groups. The blue bar and red bar in panels B and C denote the control and PE groups, respectively. * stands for p-value <0.01. ** stands for p-value <0.001.

T cells, as another major group of immune cells, were selected to perform the next round of analyses. As a result, 21 clusters were obtained by reclustering CD3+ T cells (**Figure S4**) and divided into five different superclusters: CD4+ T cells, CD8+ T cells, gamma delta T (gdT) cells, double negative T (DNT) cells, and NKT cells (**Figure 3A**). The median frequency of CD4+ T cells increased while that of CD8+ T cells decreased in PE, and their proportions were 46.69% and 35.86% in the PE group respectively, and 42.60% and 41.14% in the control group respectively, which constituted the main proportion of T cells. Following CD4+ and CD8+ T cells, gdT, NKT, and DNT cells accounted for 6.97%, 9.79% and 0.68% in the PE group respectively, and 7.38%, 7.46%, and 1.41% in the control group respectively, and showed no obvious differences between the PE and control groups (**Figure 3B**). Subsequently, subsets such as T helper 1 (Th1), Th2, and Treg cells were further divided from CD4+ T cells, accounting for 14.93%, 3.78%, and 9.81% in PE group respectively, and 10.78%, 3.93% and 5.19% in control group respectively, but no significant difference between the two groups was found. Among the subsets of CD8+ T cells, CD11c+CD8+ T cells (accounting for 0.69% and 2.06% in PE group and control group, respectively) were significantly reduced in the PE group, which indicates that this group of T cells may maintain the normal immune state in healthy pregnancies (p < 0.05) (**Figure 3C**). Although the frequency of the majority of the T cells showed no significant difference in the decidua of PE mothers, the marker expression pattern was altered, as costimulatory receptor OX40, chemokine receptors CXCR5 and CCR4, immune checkpoint Tim-3, and CD27 were all increased significantly in the PE group (p < 0.05) (**Figure 3D**).

### 3.4 A novel CD56-CD49a-CD38+ NK cell subset decreased significantly in PE

**Figure 4:**
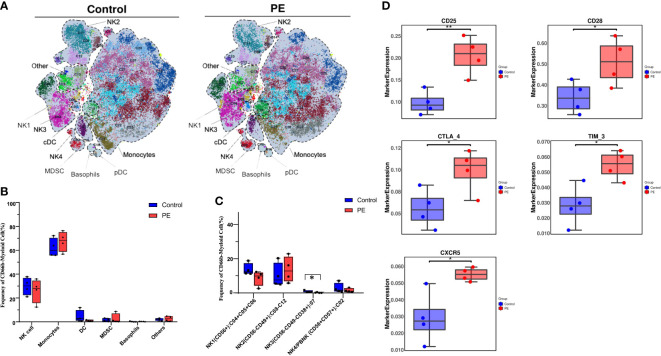
A novel CD56-CD49a-CD38+ NK cell subset was found to be decreased significantly in PE by reclustering CD45+CD3-CD19-CD66b- myeloid cells. **(A)** The main subsets of the CD45+CD3-CD19-CD66b- myeloid cells were merged as a t-SNE plot of 21 clusters of CD3+ T cells from the PE and control groups and merged as monocytes, natural killer (NK) cells, dendritic cells (DCs), myeloid-derived suppressor cells (MDSCs), basophils, and others, among which NK cells were further divided into four subsets. **(B)** Bar plot of the frequencies of the merged subsets shown in panel **(A, C)** The frequencies of the four NK cell subsets are shown in the bar plot. **(D)** The markers expressed were significantly different in NK cells of the PE and control groups. The blue bar and red bar in panels B and C denote the control and PE groups, respectively. * stands for p-value <0.01. ** stands for p-value <0.001.

A reclustering analysis of myeloid populations was also carried out in this study. The CD45+CD3-CD19-CD66b- populations were reclustered into 28 clusters (**Figure S3**), which were merged into six superclusters (**Figure 4A**). Monocytes and NK cells were the main superclusters in this population, accounting for 67.20% and 26.72% in PE group respectively, and 63.69% and 28.74% in control group respectively. The remaining cells were dendritic cells (DCs), myeloid-derived suppressor cells (MDSCs), basophils, and others. Monocytes increased slightly in the PE group, but no significant difference was found between the two groups (**Figure 4B**). NK cells, as one of the main cell type proportions among decidual myeloid cells during delivery, were ultimately separated into four subsets according to the expression of key markers: NK1 (CD56+CD49a+), NK2 (CD56-CD49a^mid/hi^), NK3 (CD56-CD49a-CD38+), and NK4/PBNK (CD56+CD57+), which accounted for 9.09%, 13.75%, 0.12% and 1.47% in PE group respectively, and 13.68%, 9.96%, 0.93% and 2.40% in control group respectively. The subset NK3, a novel identified NK cell group, decreased significantly in the PE group (p < 0.05) (**Figure 4C**). The expression of CD25, CD28, CTLA-4, TIM-3, and CXCR5 was significantly upregulated in the PE group (**Figure 4D**).

## 4 Discussion

In the current study, decidual immune cells from PE patients and normal parturients were characterized by CyTOF and clustered into six superclusters: NK cells, T cells, B cells, monocytes, granulocytes, and others. Among these six superclusters, only the proportion of B cells significantly differed between the PE and control groups and was significantly decreased in the PE group, which suggests that B cells may play an important role in the development of PE. Although a few studies on PE conducted two decades ago supported that B cells were involved in PE, recent studies have also gradually pointed out that B cells constitute a dominant element in the pathogenesis of PE; the activation status of B cells may represent a valid therapeutic approach in PE, and B cells have been identified as a target because of their immune modulatory action (28). However, the phenotypic and functional changes of decidual B cells in PE have rarely been explored, and their value in measuring at delivery in the early pathogenesis of PE is unclear. In our study, it was found that CD27+CD38+ Breg-like cells were obviously decreased in PE, which contributed most to the statistical significance of the total B-cell reduction in PE, although no significant difference was detected. Studies found that during pregnancy in humans, Bregs (CD24+CD27+ B cells) in peripheral blood are significantly higher than those in women who are not pregnant or in women with spontaneous abortions (29), and other Bregs (CD24+CD27+CD38+ B cells) are reduced in the third trimester of pregnancy and on the day of delivery compared with nonpregnant control women, which may be due to the cessation of the activation of sex hormones activating B cells (30). Bregs are a potent source of interleukin IL-10, which is an anti-inflammatory cytokine that is essential for positive pregnancy outcomes, and its deficiency may lead to fetal growth restriction, resorption, and even fetal death (31). A suppressive function for B cells was first postulated in the 1970s by the observation that B cell-depleted splenocytes were unable to suppress delayed-type hypersensitivity in guinea pigs upon adoptive transfer (32), and this notion was further confirmed in B cell-deficient mice that were unable to recover from experimental autoimmune encephalitis (EAE) (33). Bregs have heterogeneous phenotypes that are activated by metabolic controlled context-dependent immune signaling pathways (34), express different transcription factors, and exert their immunosuppressive function through direct contact and IL-10, transforming growth factor beta (TGF-β), and other immunologic factors. This means that B cells might potentially differentiate into Bregs in response to the right environmental stimuli. To our knowledge, decidual B-cell subsets have not yet been characterized in PE and healthy pregnant women. We infer that CD27+CD38+ cells may be a specific subset of Bregs in the decidua in the third trimester of pregnancy that was first uncovered in this study. We also found that a cluster of CCR6+CCR4+CXCR3+CTLA-4+ migrating B cells increased significantly in PE, and the chemokine CXCR5 was significantly increased in B-cell superclusters. CXCR5 is an essential contributor to B-cell proliferation, differentiation, and high-affinity antibody synthesis (35, 36). Overexpression of CXCR5 on B cells leads to overactivation of B cells and then causes antibody-mediated autoimmune diseases. We infer that the high expression of CXCR5 causes B-cell overactivation and massive chemotaxis to the decidua, resulting in the production of excessive autoantibodies and the pathogenesis of PE.

In addition to B cells, T cells are another indispensable group of immune cells involved in the development of PE. A systemic and local imbalance between Tregs and effector T cells, which stimulates Th1 activation and subsequently elevates the Th1/Th2 ratio and results in a pro-inflammatory state, was also found in PE (37, 38). Tregs are critical for establishing a receptive decidual environment and placental development, and their depletion in early pregnancy will cause PE and fetal loss (9, 39). Although no significant change in the total T cells in the decidua was detected between PE patients and healthy parturients in this study, a special group of CD11c+CD8+ T cells was found to be significantly decreased in PE patients. CD11c+CD8+ T cells represent an important category of Tregs (40) and seemingly function as “regulatory” or “effector” cells dictated by the microenvironment of disease-induced inflammation (41). The expansion of CD11c+CD8+ T cells could induce DCs and macrophages to derive immunosuppressive IDO and further suppress pathogenic CD4+ T cells (42). Therefore, the lack of CD11c+CD8+ T cells may contribute to PE development because of the runaway pro-inflammatory effect of CD4+ T cells and sustained immune activation. On the other hand, CD11c+ CD8+ T cells could also function as an effector depending on the surrounding environment that boosts immune potential and halts the progression of cancer and other immune-related diseases (43, 44). Hence, metabolic disorders may cause an imbalance in total T cells by decreasing CD11c+CD8+ T cells and may further lead to the pathogenesis of PE.

NK cells are recruited and activated by ovarian hormones and have pivotal roles throughout pregnancy. Previous studies have illustrated that in either mouse models or humans, the reduction in NK cells is associated with intrauterine growth restriction (IUGR), gestational hypertension, or a high uterine artery resistance index (45, 46). Consistently, NK cells also showed a reduction trend in PE patients in our study. In addition, the subsets of NK cells at the maternal–fetal interface were revealed by single-cell sequencing, and it was found that most tissue-resident NK (trNK) cells are a subset of CD56bright NK cells (47). Our study found that a subset of CD56-CD49a-CD38+ NK cells decreased significantly in the PE group, which accounting for 0.49% and 3.44% in PE and control groups, respectively. CD38 is a hydrolase of nicotinamide adenine dinucleotide (NAD) involved in cellular metabolism (48). CD38^KO^ NK cells have a unique metabolic reprogramming with a high mitochondrial respiratory capacity (49). Adaptive NK cells mediate antibody-dependent cellular cytotoxicity (ADCC) in multiple myeloma and can be improved by treatment with anti-CD38 mAbs, such as daratumumab and isatuximab (50). MDSCs, which express CD38, were selected for the evaluation of immunosuppression (51). It was speculated that this group of suppressive NK cells may develop specifically under the placental physiological hypoxic environment. The significant deletion of this unique group of NK cells in PE may lead to the failure to suppress inflammation at the maternal–fetal interface during PE progression.

In addition to the phenotype of CD45+ immune cells, we checked the functional markers expressed in each supercluster. OX40 and CXCR5 were significantly increased in the PE group and were especially significantly increased in T- and B-cell superclusters. OX40 is transiently expressed on CD4+ T cells after 12–24 h of activation, which upregulates the CXCR5 expression (52). The significant upregulation of OX40 and CXCR5 prompts autoantibody production in B cells, which is implicated in the pathogenesis of PE. Recently, a study pointed out that the messenger RNA (mRNA) expression of CXCR5 was significantly upregulated in the peripheral blood of PE patients (53). The upregulation of OX40 and CXCR5 in T- and B-cell superclusters and total decidual immune cells in PE implied that the action of T and B cells in the decidua promotes the production of autoantibodies and participates in the occurrence of PE.

Our research was a worthy attempt to investigate the phenotype and function of CD45+ immune cells in the decidua of PE patients, which contributes to understand the pathogenesis of PE, and the altered cell subsets and markers may inspire the immune modulatory therapy for PE. However, the limitations of this study should also be addressed. First, the sample size was relatively small, and insufficient data may not be able to identify accurate significant relationships between PE and healthy pregnancies. Validation research with a larger sample size should be performed in future studies. Second, the cells discovered in this study, especially Bregs and Tregs, have metabolism-dependent functional features. Hence, metabolome analysis should be carried out next to construct a comprehensive system of immune cell interactions. Moreover, all of the outcomes were obtained from mass cytometry data analysis, and specific cells should be isolated and studied by benchworks to verify their roles in the pathogenesis of PE.

## Data availability statement

The original contributions presented in the study are included in the article/**Supplementary material**. Further inquiries can be directed to the corresponding author.

## Ethics statement

The studies involving human participants were reviewed and approved by Research Ethics Committee of Peking University Shenzhen Hospital. The patients/participants provided their written informed consent to participate in this study.

## Author contributions

SF, CL, HW, XLL, and XZ designed the study. CL, SZ, HW, ZX, and XW collected the samples. SZ, YL, PL, and LH collected the clinical data and performed the experiments. XZ, MF, JL, and XYL analyzed the data. XZ and JL organized the figures and tables. SF, MF, and XZ wrote the manuscript. SF, XZ, YL, XLL, and HW were responsible for the funding support. All authors contributed to the article and approved the submitted version.
