# Supplementary material for: Phenotypic and functional alteration of CD45+ immune cells in the decidua of preeclampsia patients analyzed by mass cytometry (CyTOF)
Source: Front Immunol. 2023 Jan 6;13:1047986. doi: 10.3389/fimmu.2022.1047986 (PMC9852836; doi:10.3389/fimmu.2022.1047986)

12474-89-CD45

	1：100	1：200	1：400	1：200
0	1	2	3	+

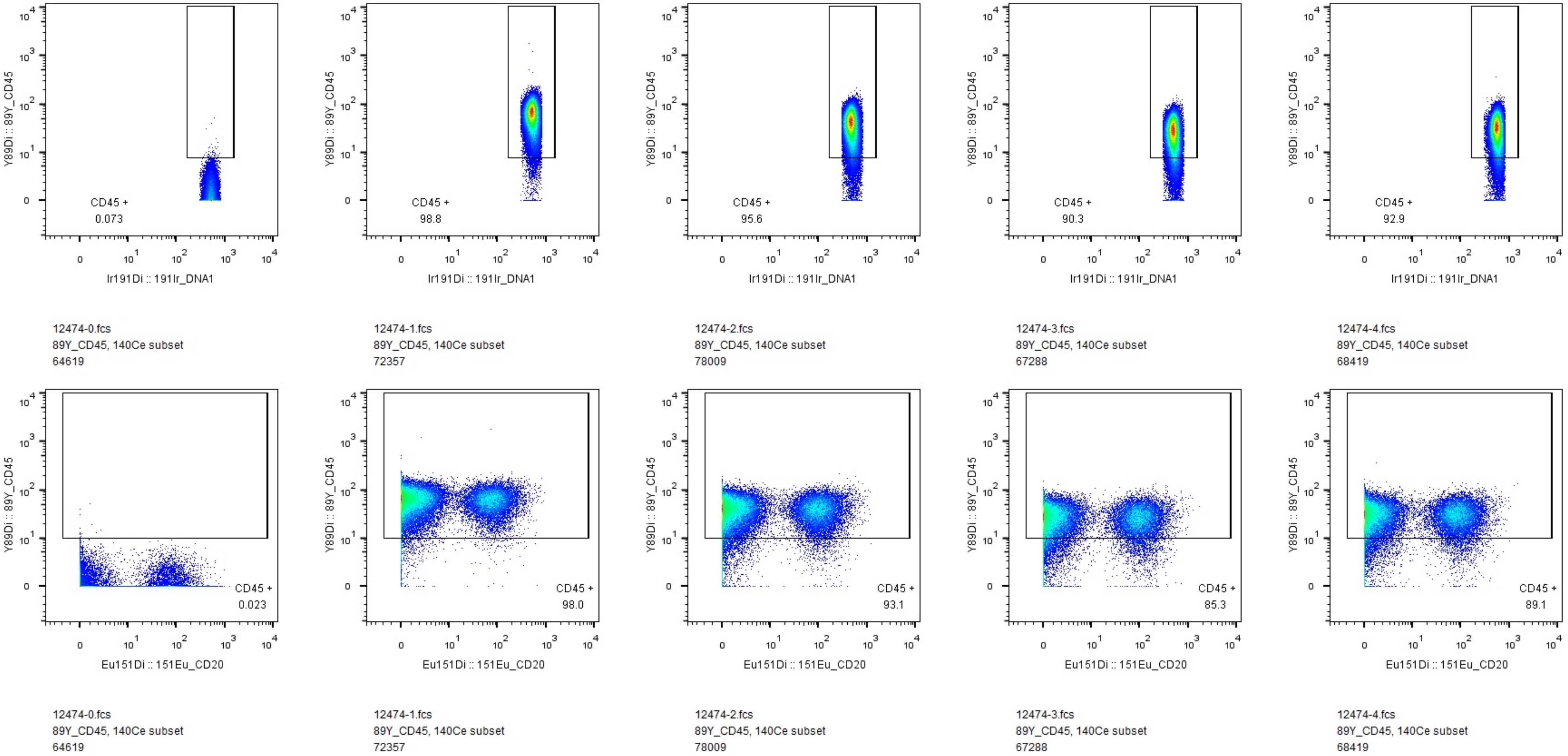

12240-115-CD3

1：50 1：100 1：200 1：50

0 1 2 3 +

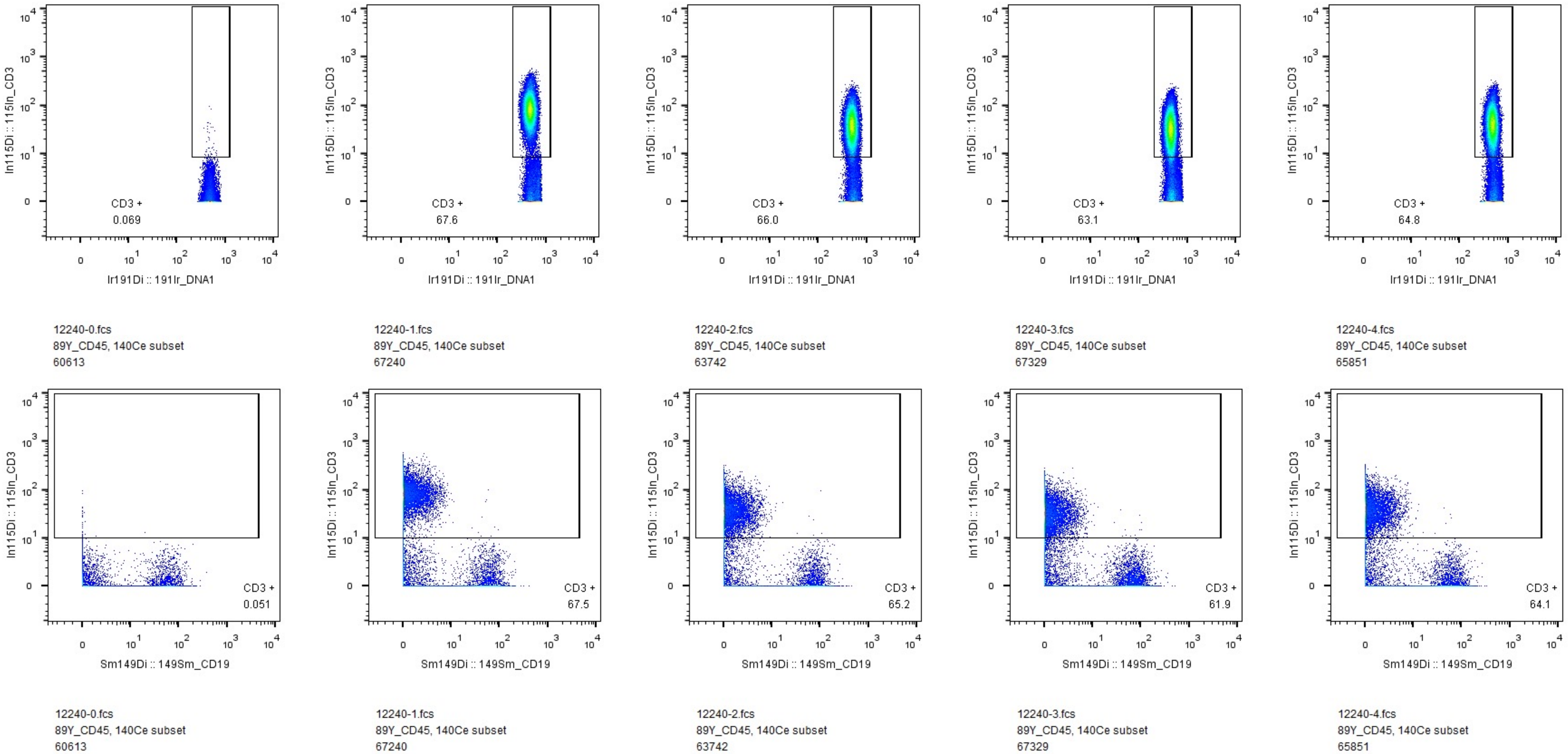

5502-139-CD68

	1：50	1：100	1：200	1：100
**0**	**1**	**2**	**3**	**+**

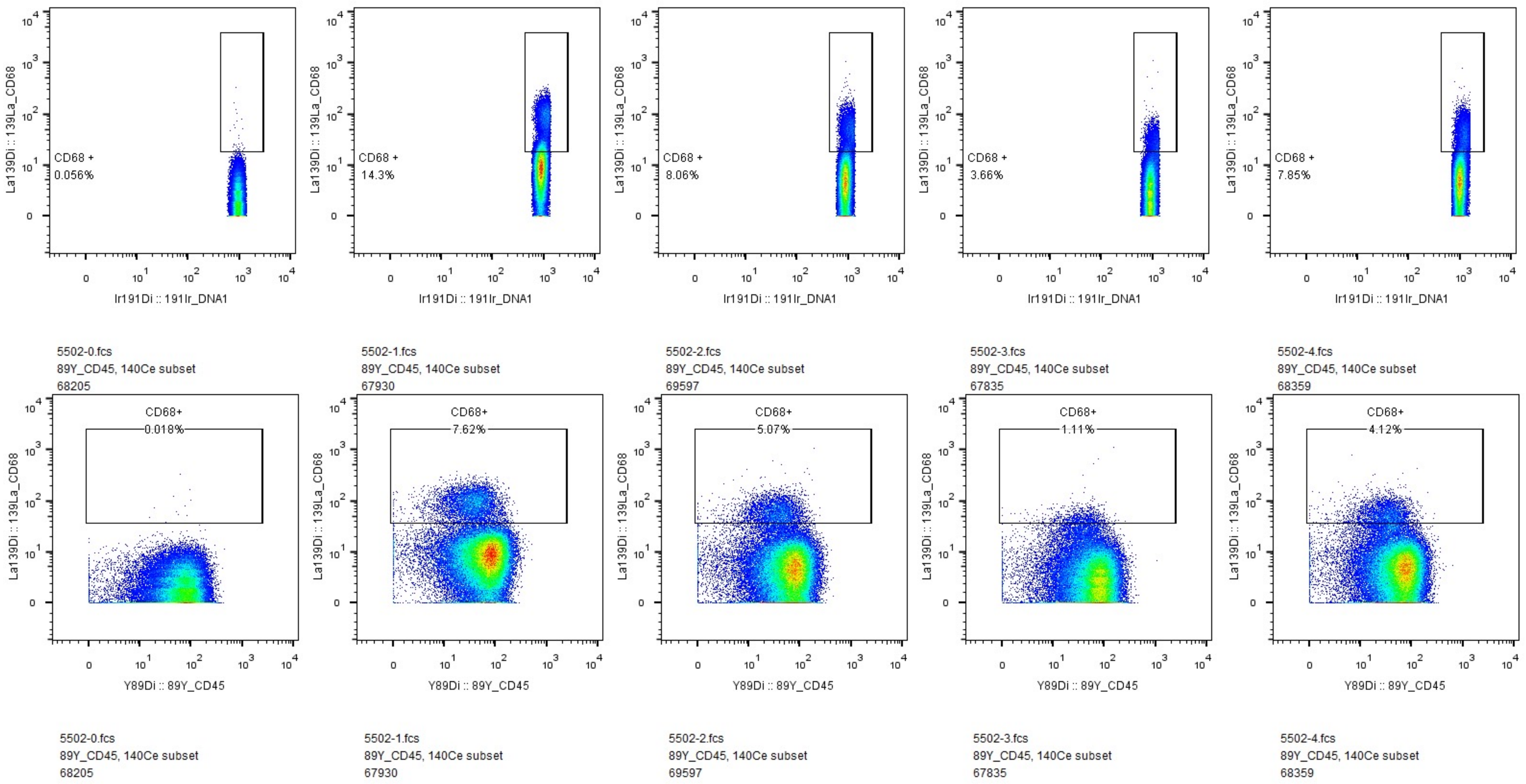

11798-141-CD56

1 : 100	1 : 200	1 : 400	1 : 200

0 1 2 3 +

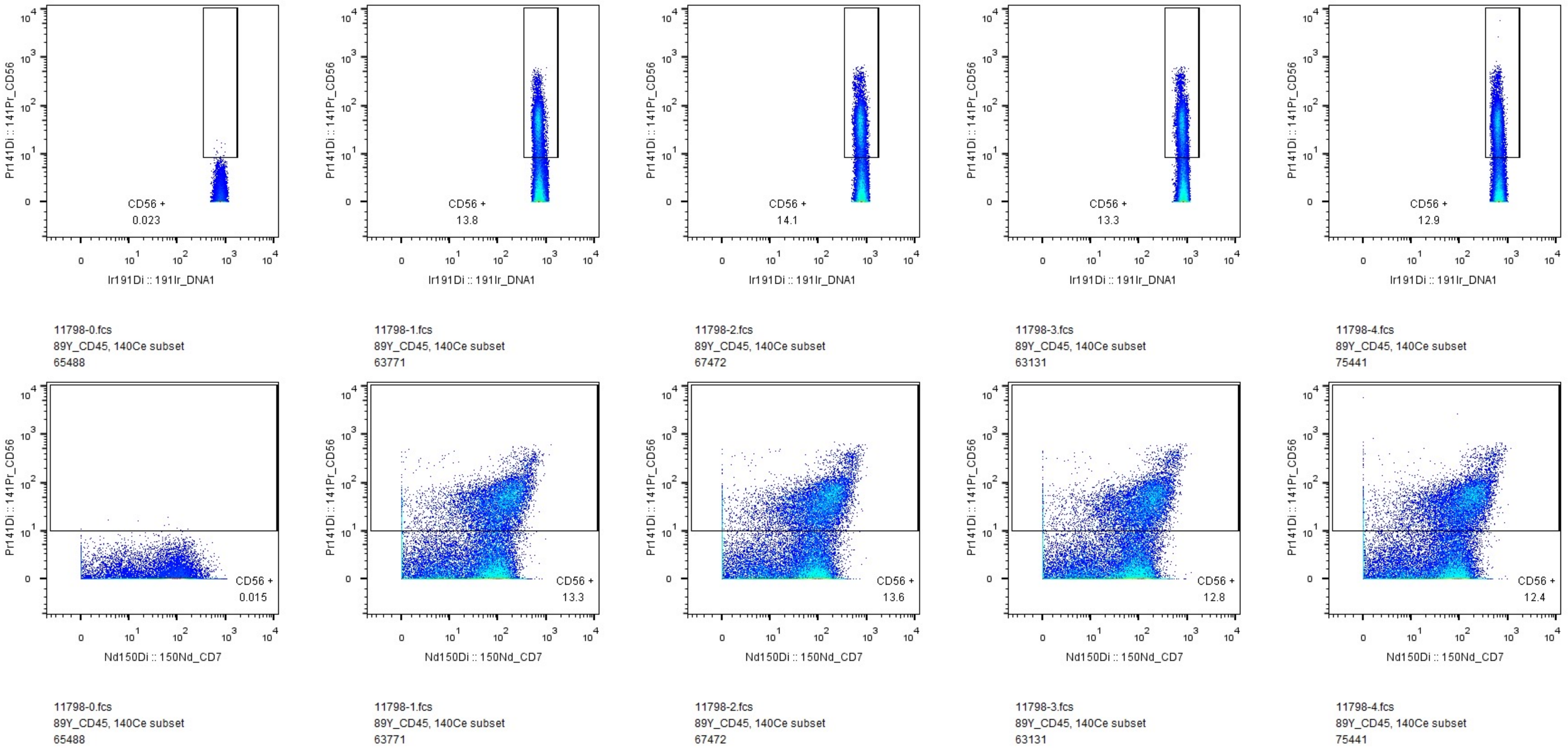

7562-142-gdTCR

1：25　　1：50　　1：100　　1：50

0　　1　　2　　3　　+

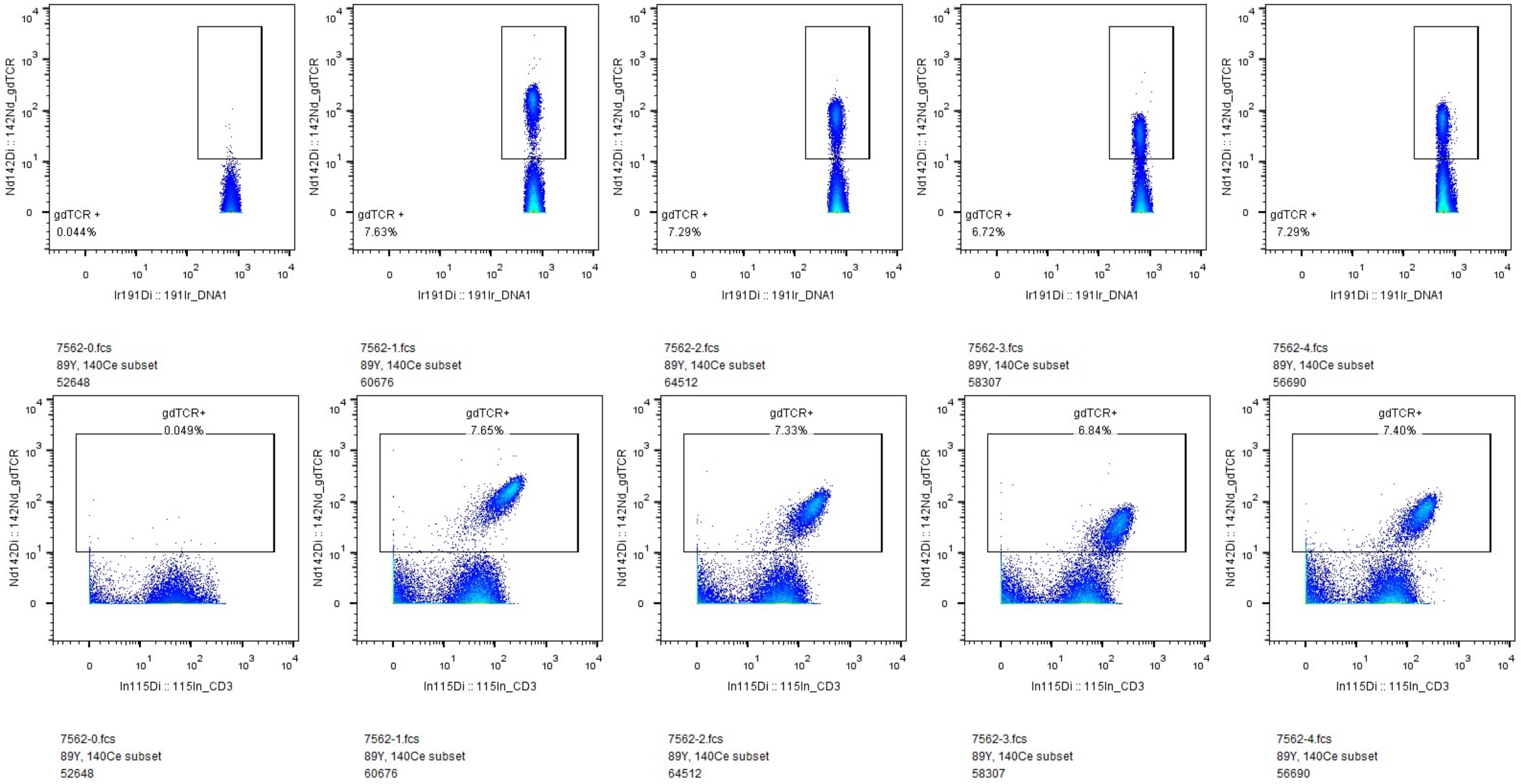

12664-143-CD196-CCR6

	1：50	1：100	1：200
0	1	2	+

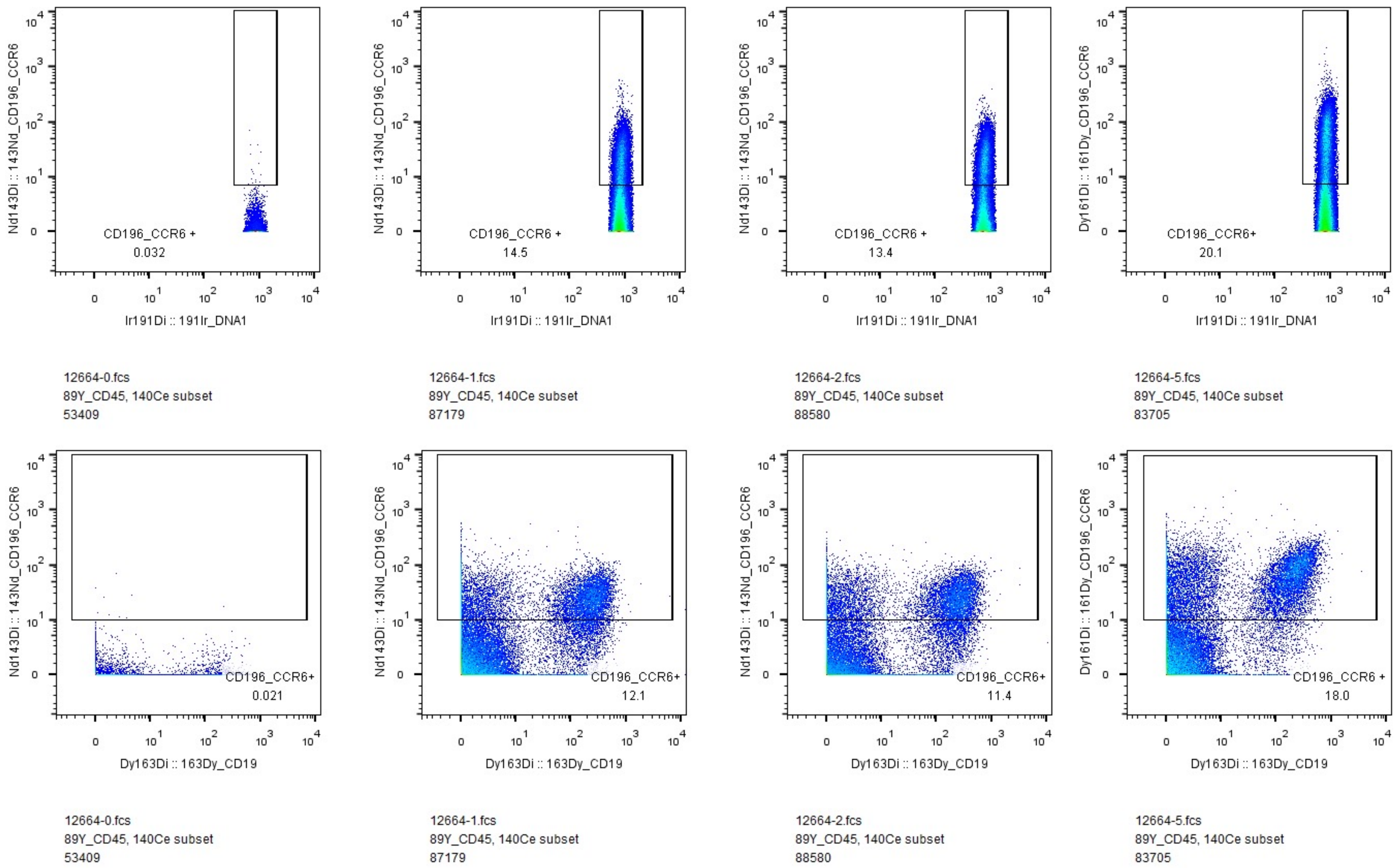

12633-144-CD14

	1 : 50	1 : 100	1 : 200	1 : 100
0	1	2	3	+

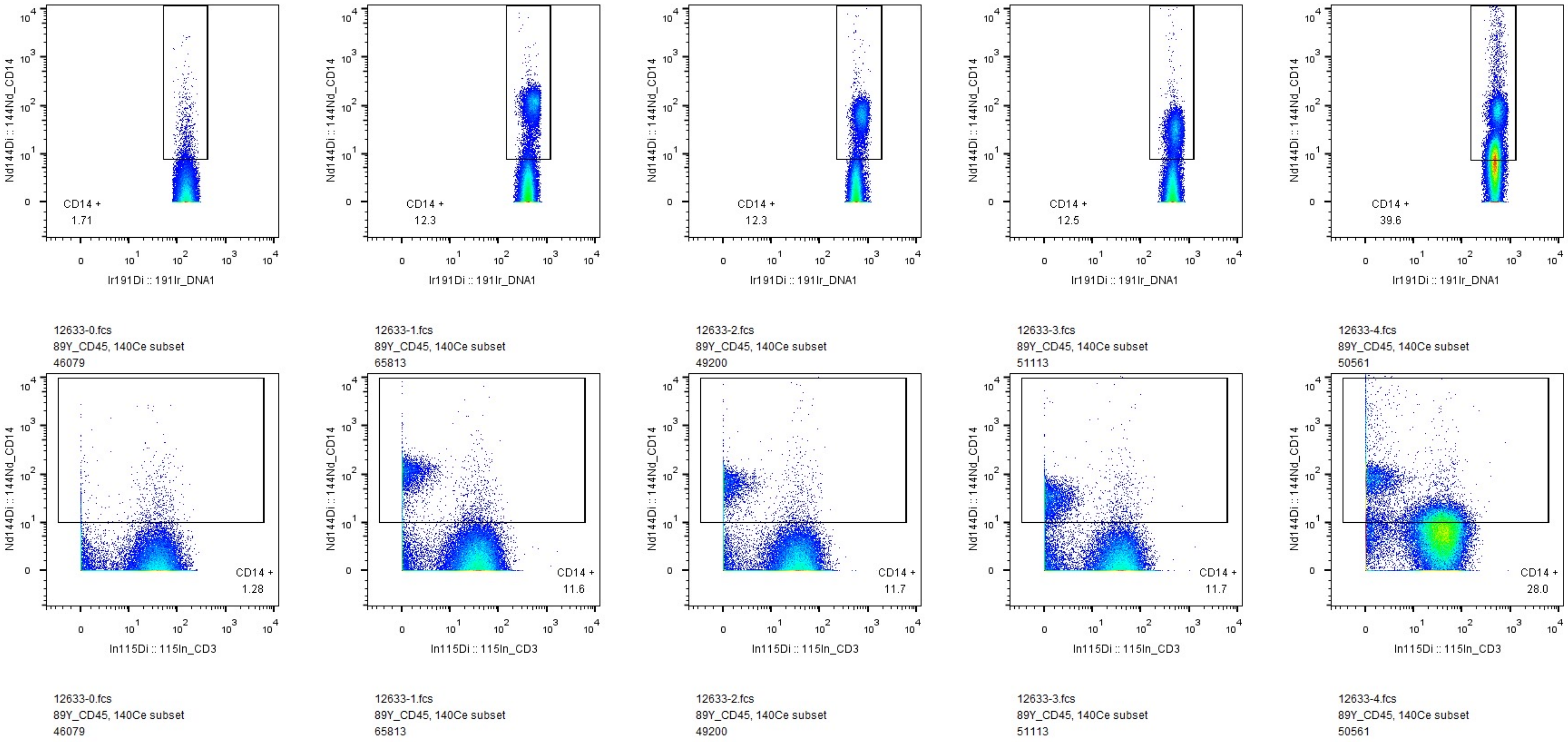

10833-145-CD103-Integrin-alphaE

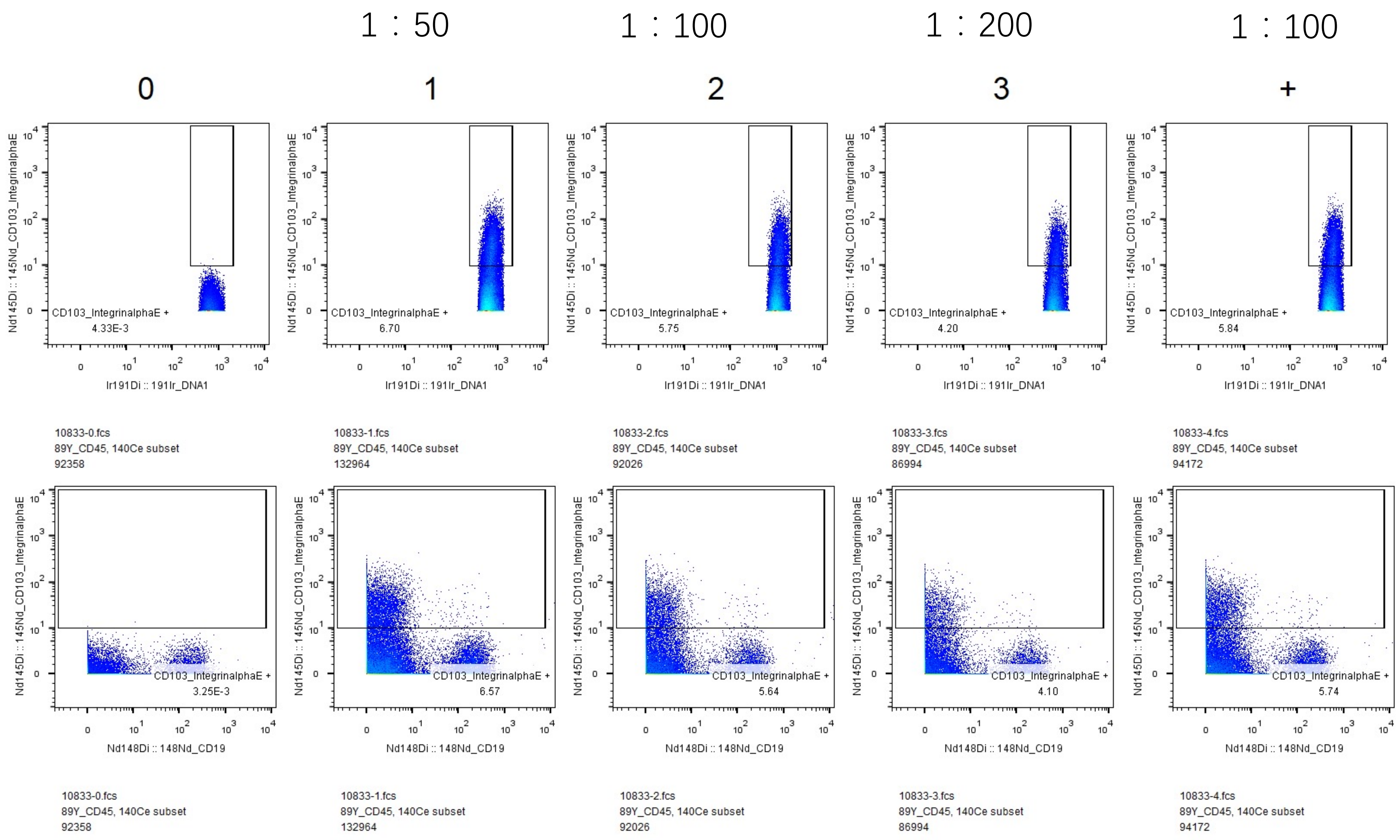

12378-146-CD123-IL-3Ra

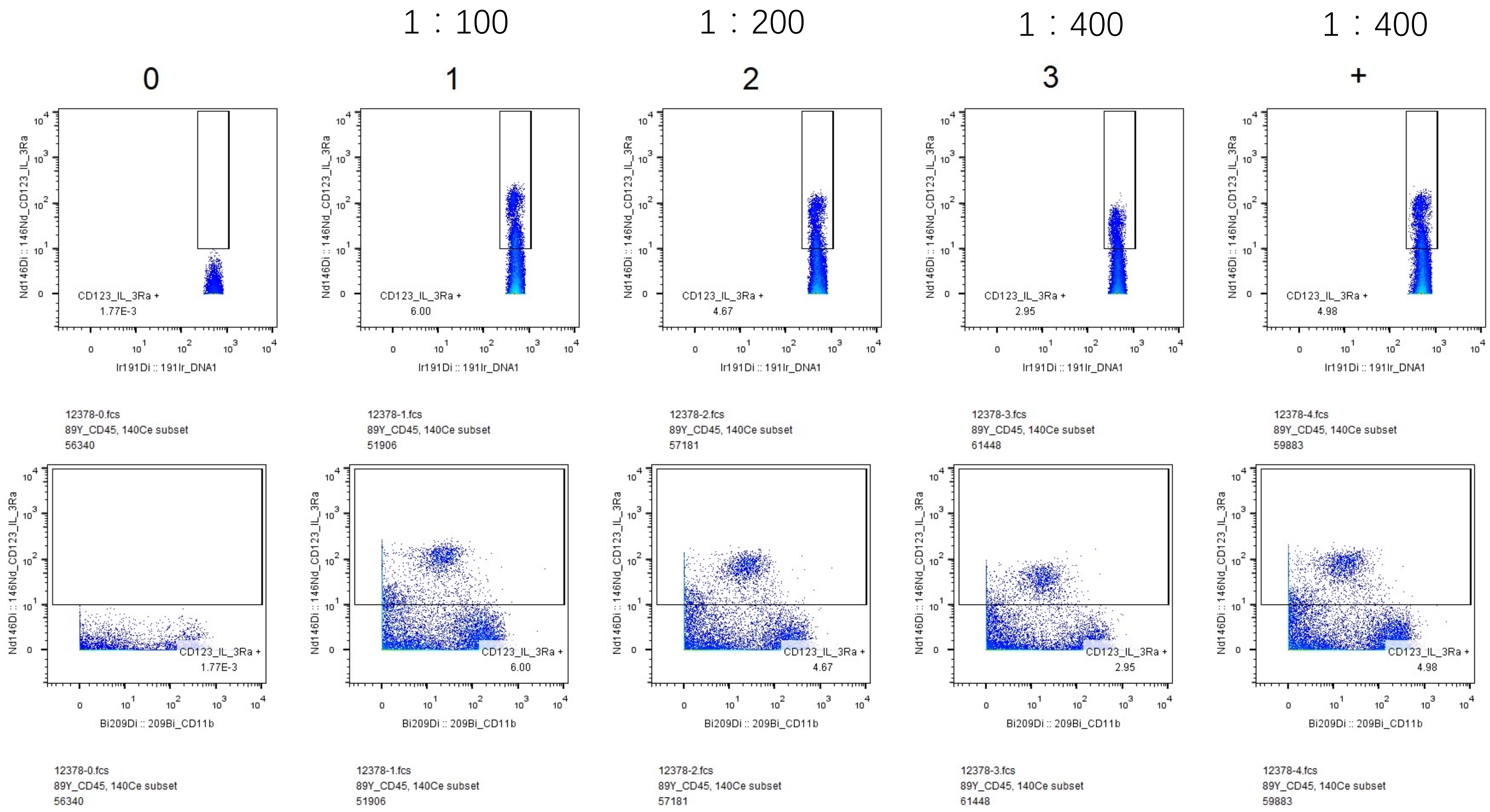

12148-147-CD366-Tim-3

	1：50	1：100	1：100
0	1	2	+

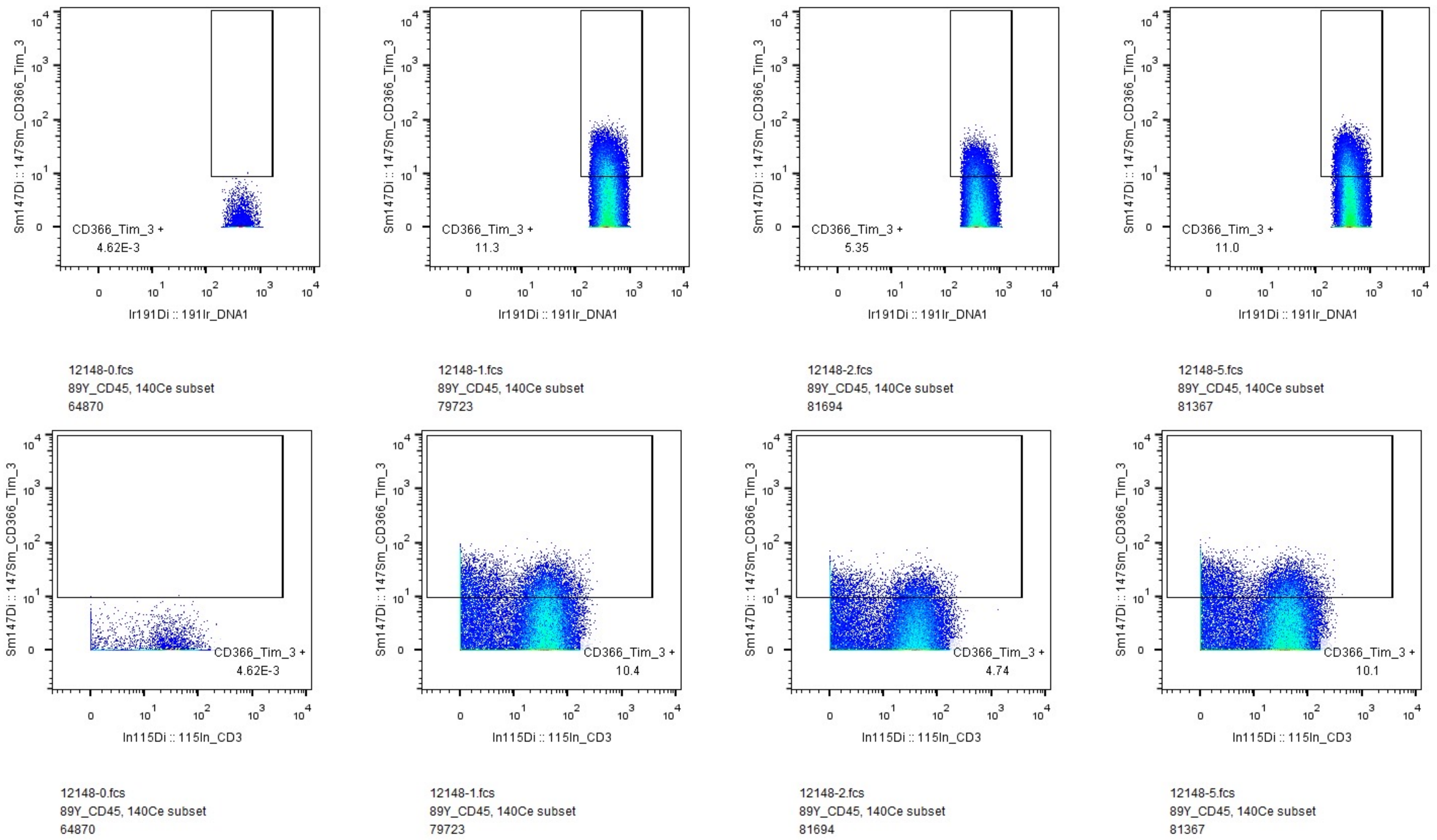

4894-148-CD28

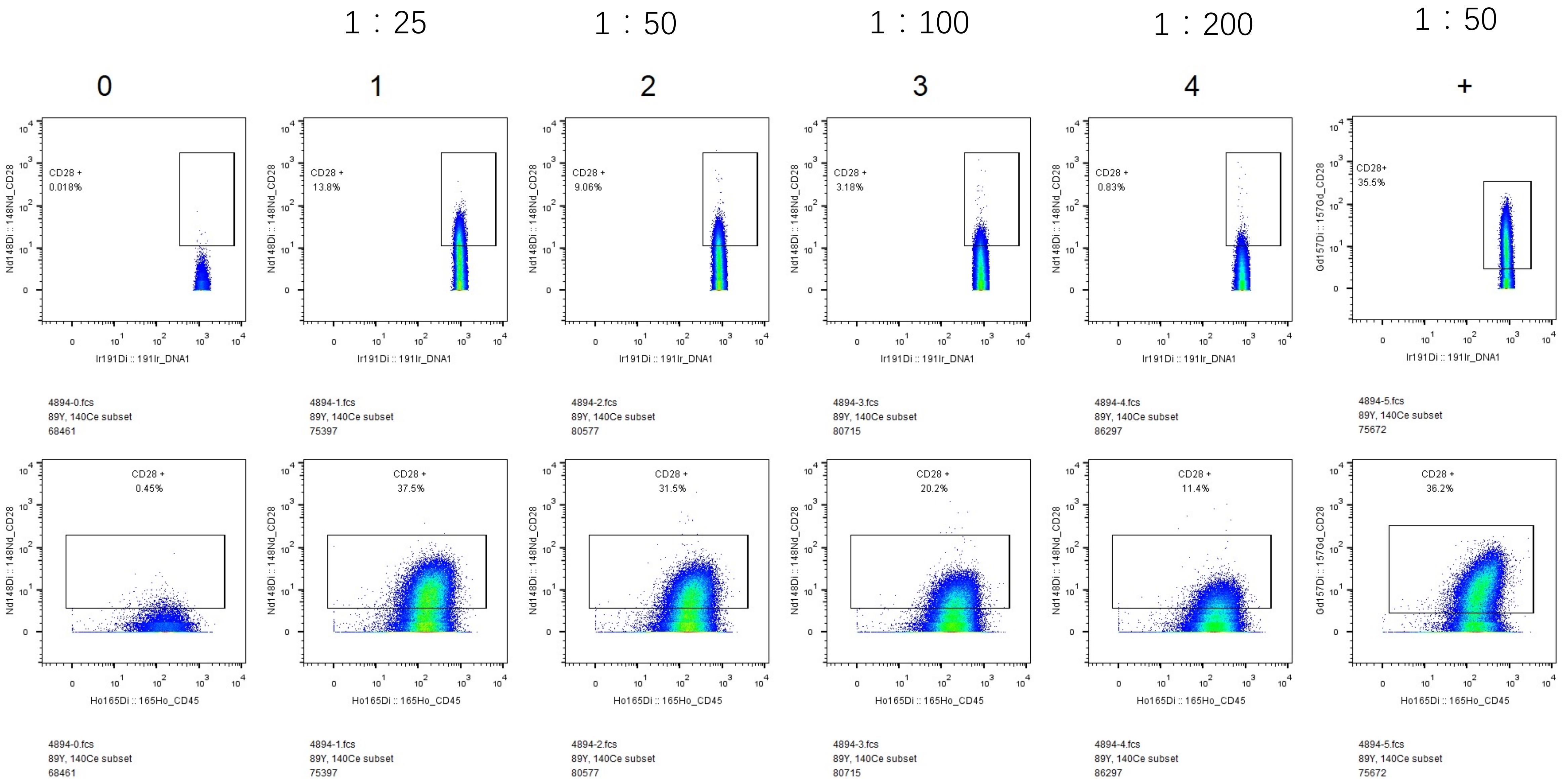

9394-149-CD25

1：100　1：200　1：400　1：800

0　1　2　3　+

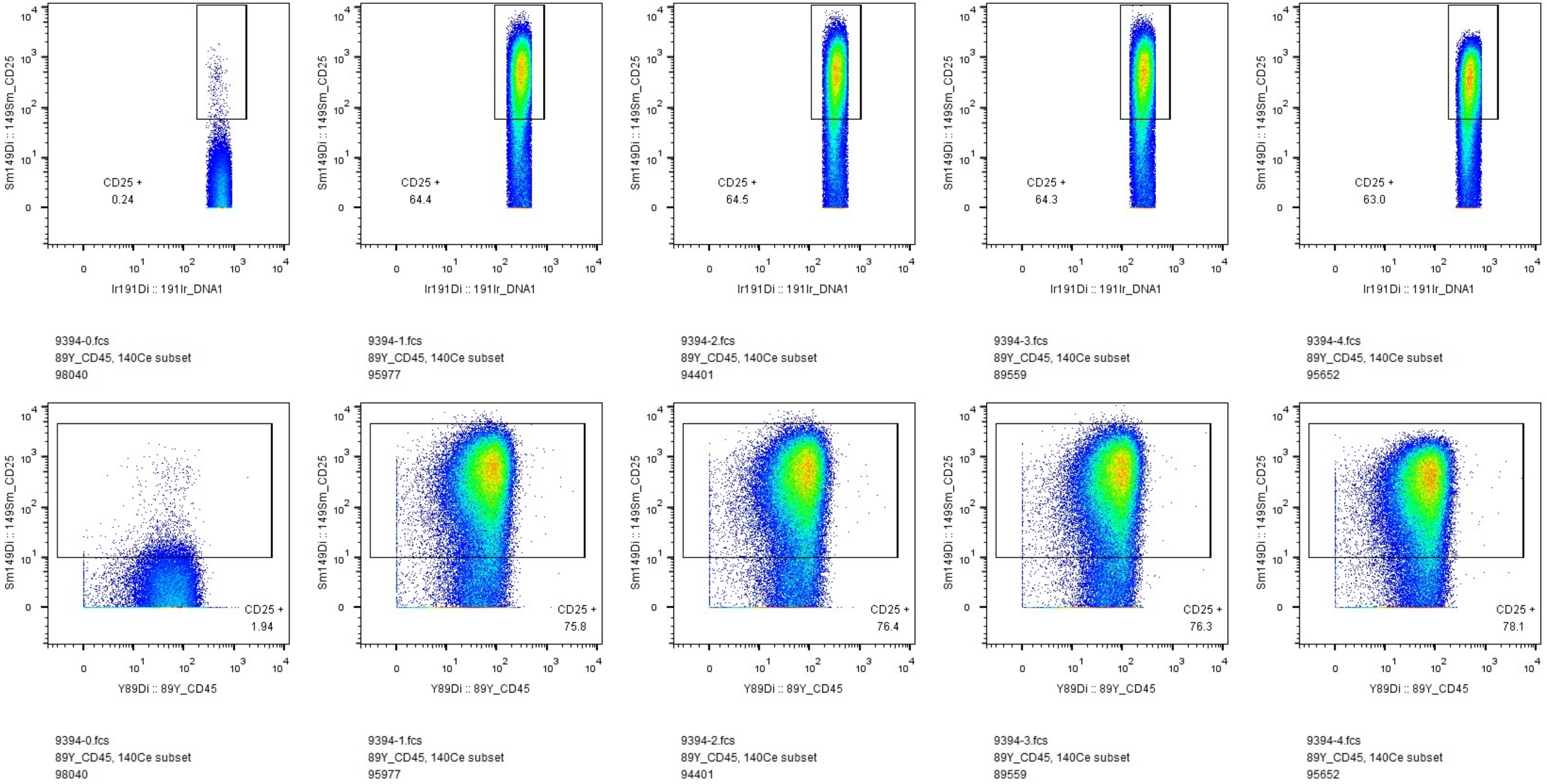

8774-150-CD73

0	1	2	3	+
	1：50	1：100	1：200	1：50

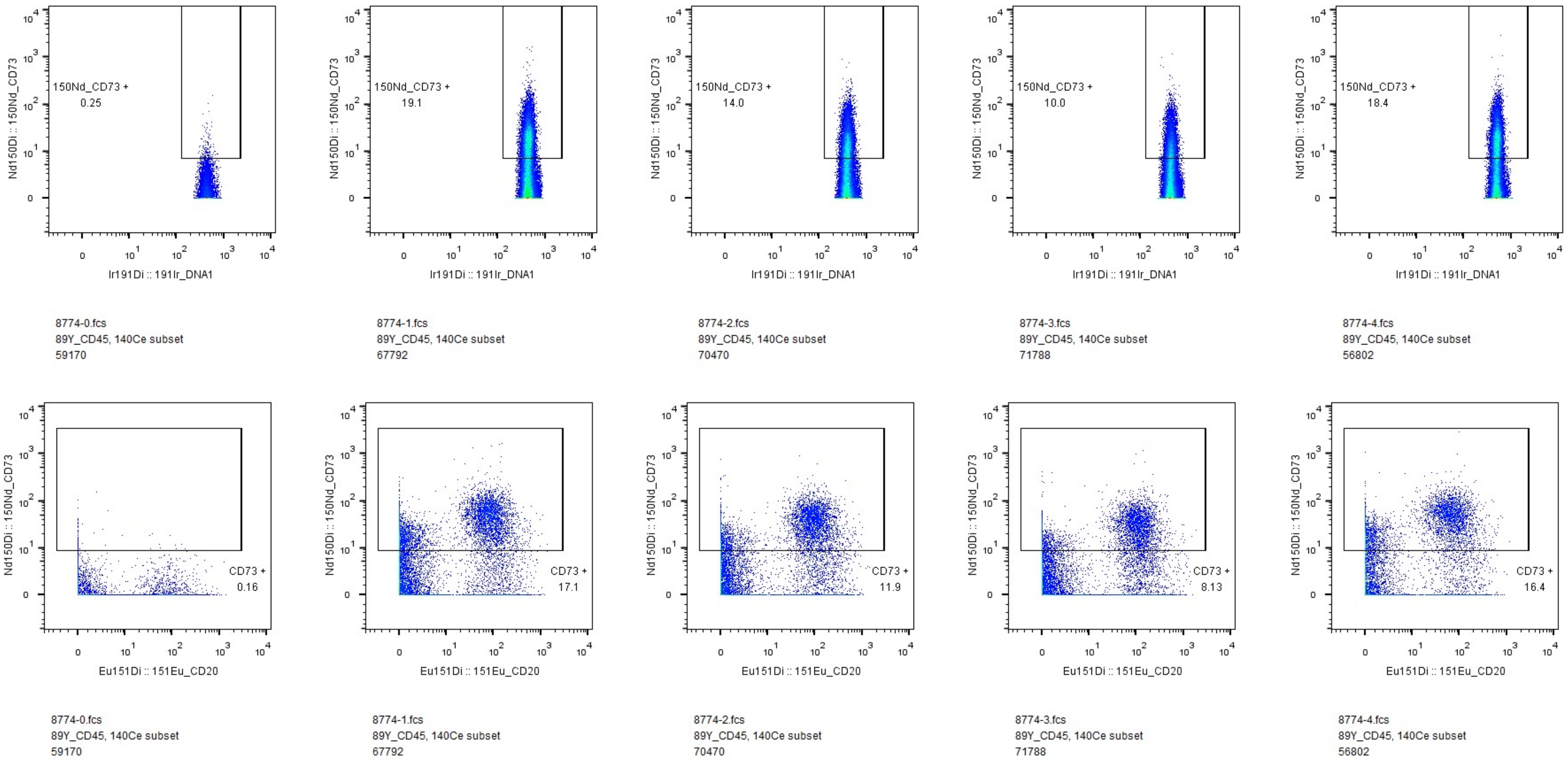

10209-151-CD158I-KIR2DS4

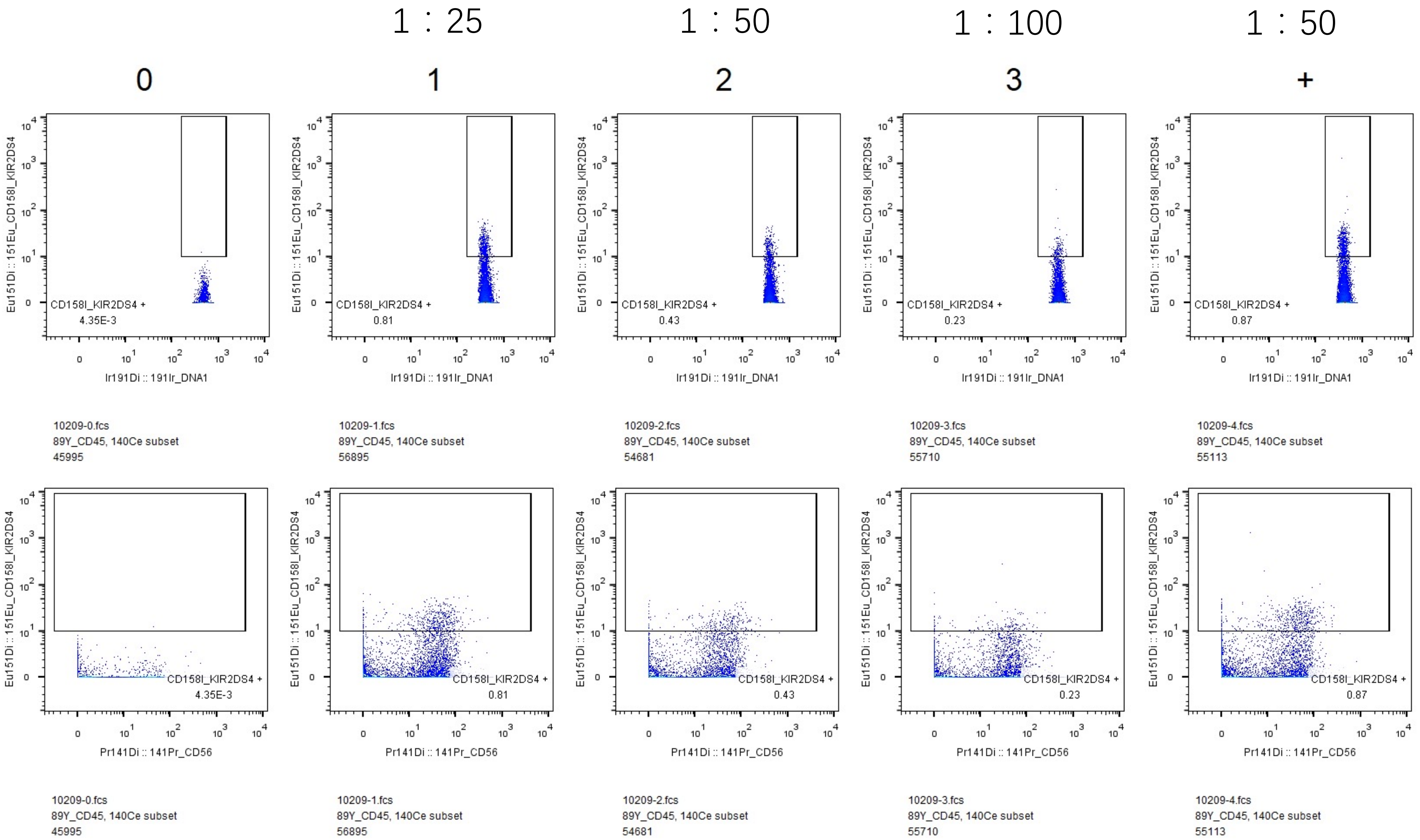

12318-152-CD223-LAG-3

	1：50	1：100	1：200	1：100
0	1	2	3	+

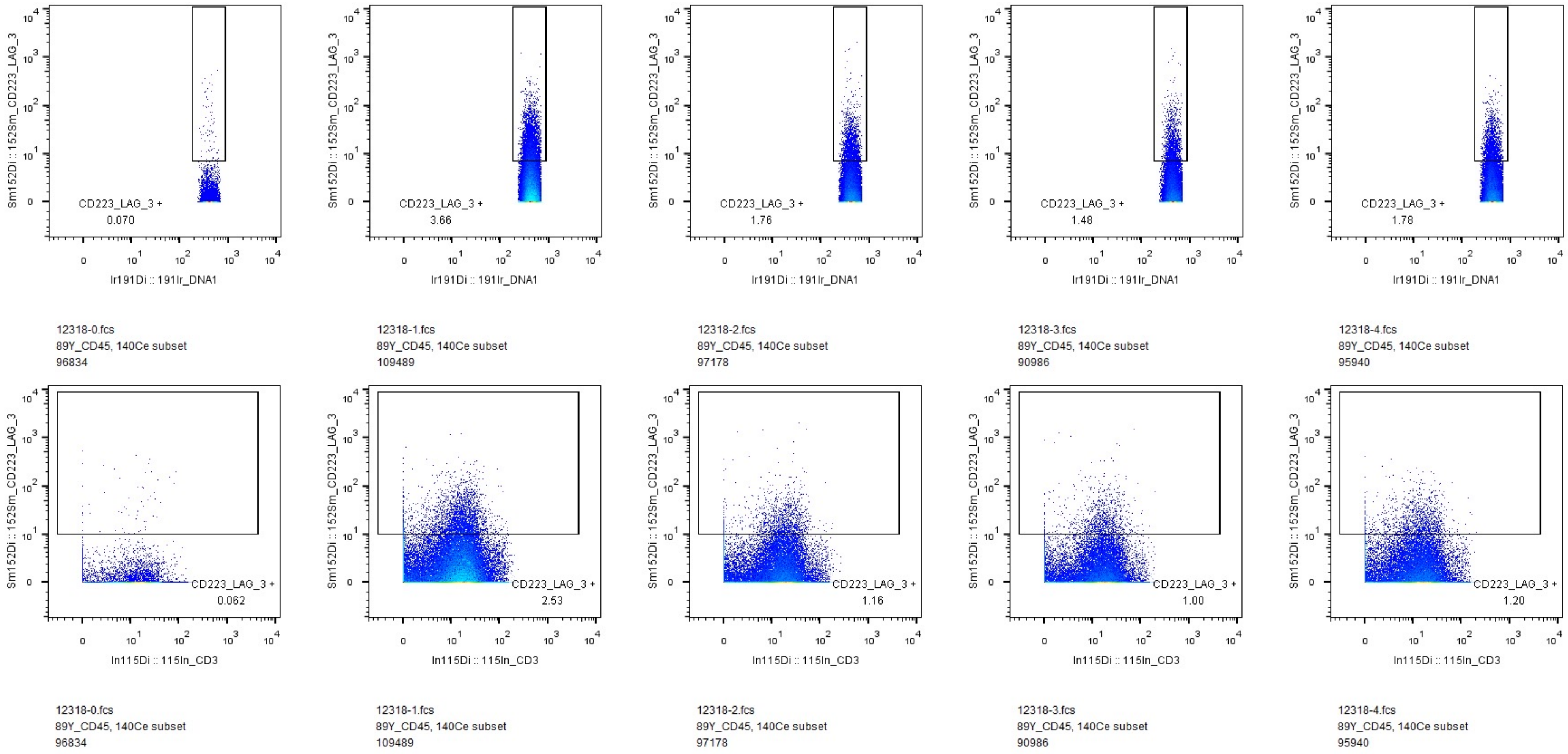

12472-153-CD57

	1：200	1：400	1：800	1：800
**0**	**1**	**2**	**3**	**+**

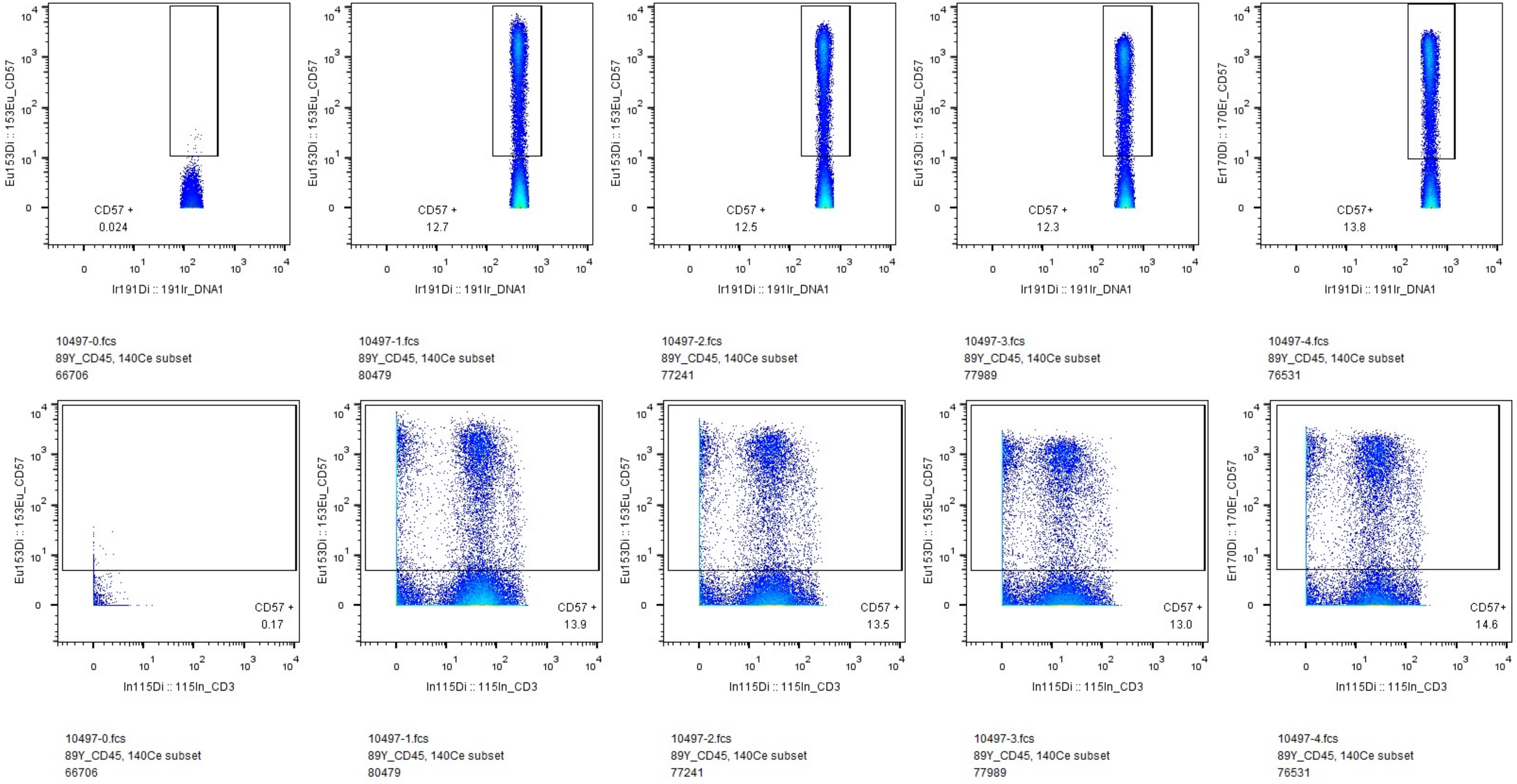

6735-154-TIGIT

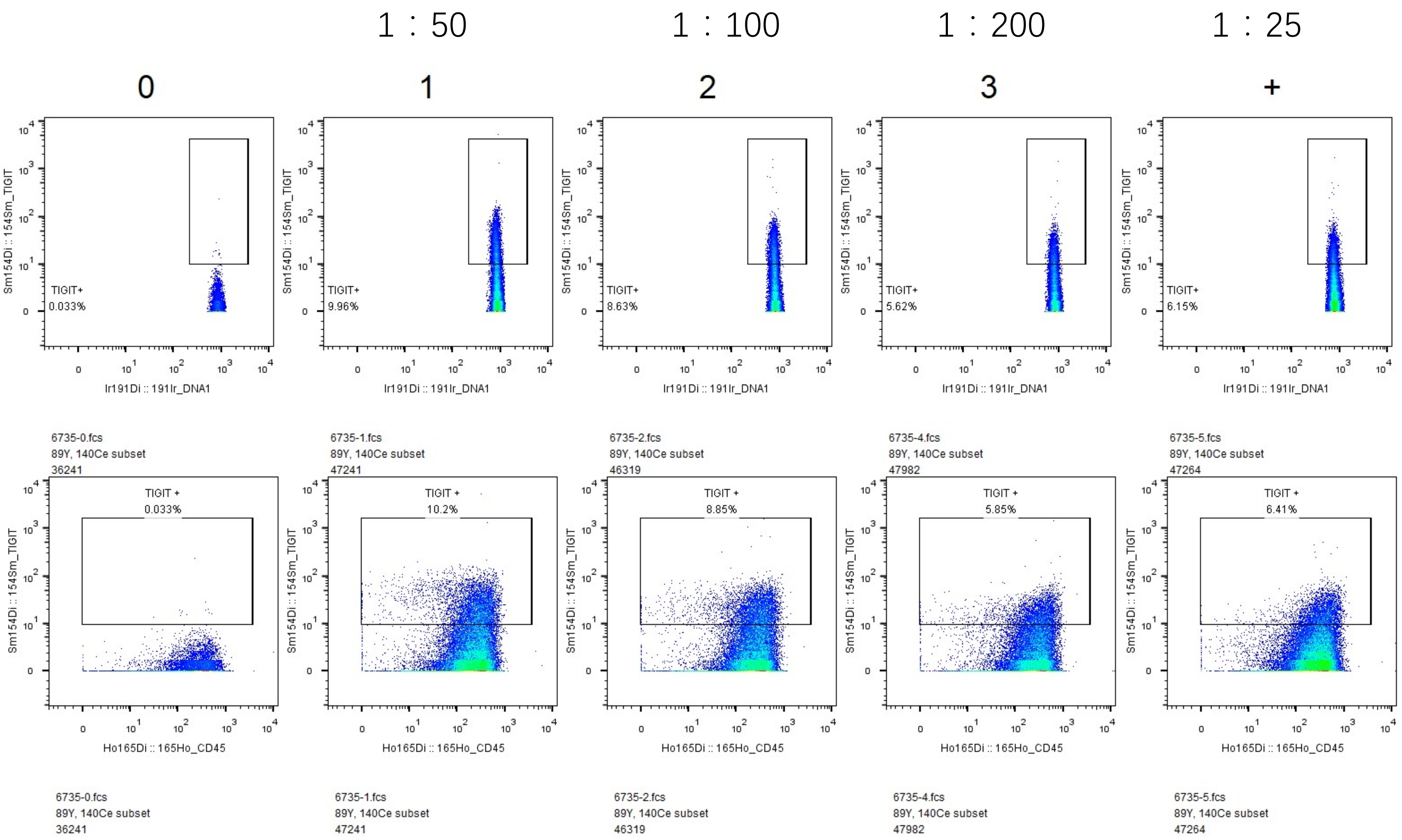

12520-155-CD49a

0

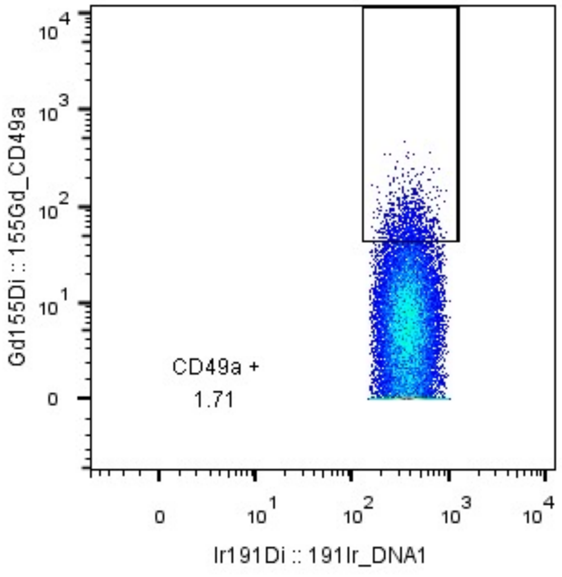

12520-0.fcs
89Y, 140Ce subset
47591

1 : 100

1

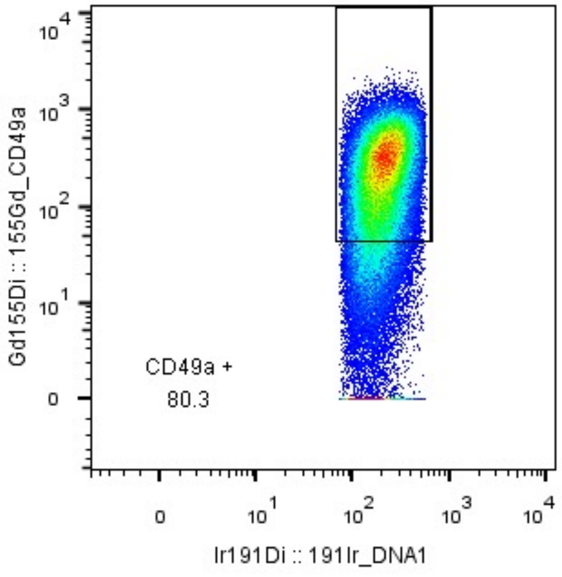

12520-1.fcs
89Y, 140Ce subset
88993

1 : 200

2

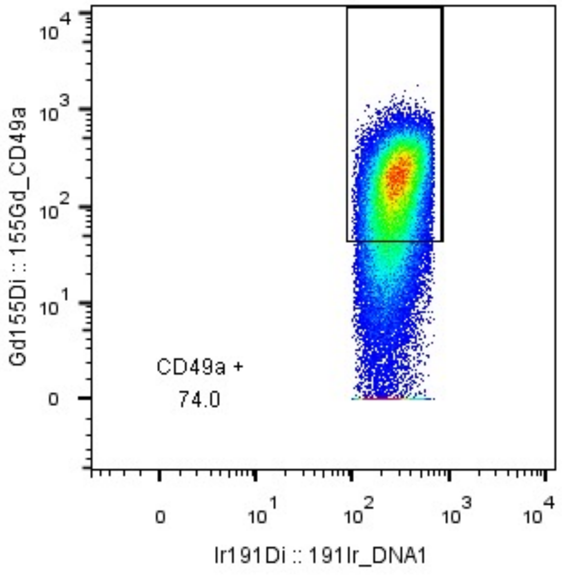

12520-2.fcs
89Y, 140Ce subset
67743

1 : 200

+

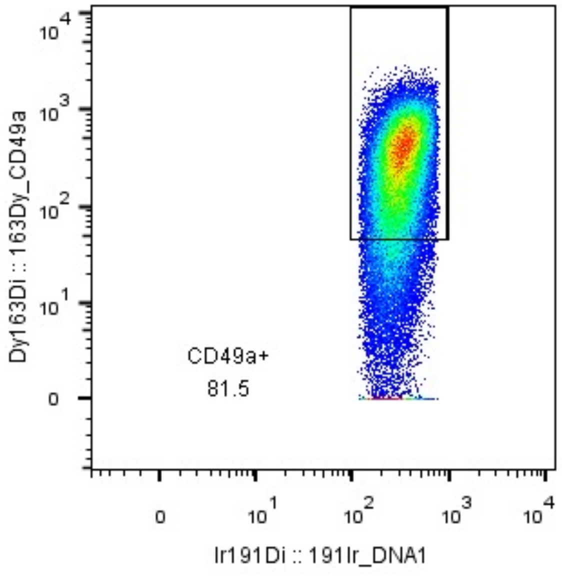

12520-5.fcs
89Y, 140Ce subset
57721

9326-156-CD194-CCR4

0	1	2	3	+
	1：100	1：200	1：400	1：200

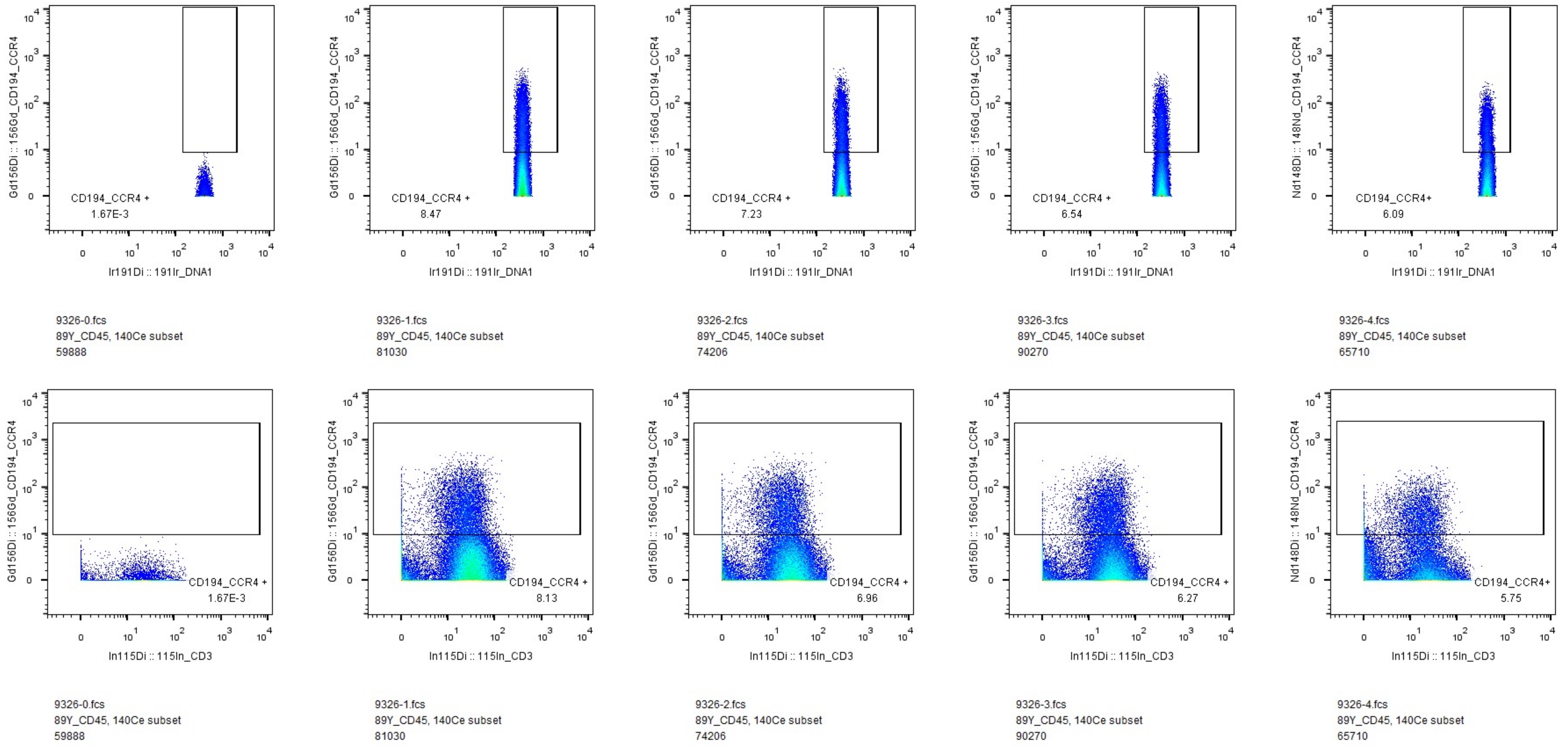

7539-157-CD274-PD-L1

	1：100	1：200	1：400	1：400
0	1	2	3	+

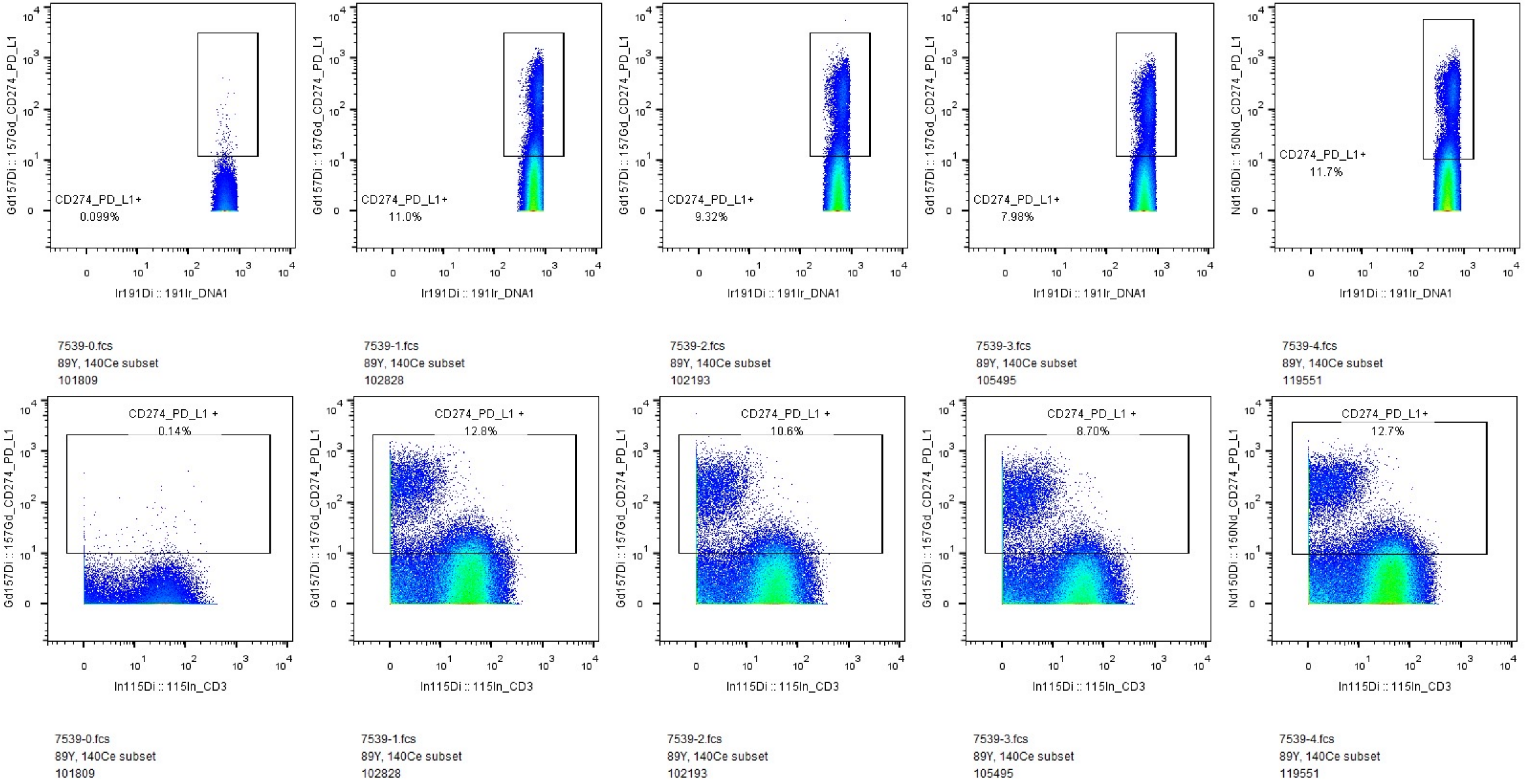

12261-158-CD197-CCR7

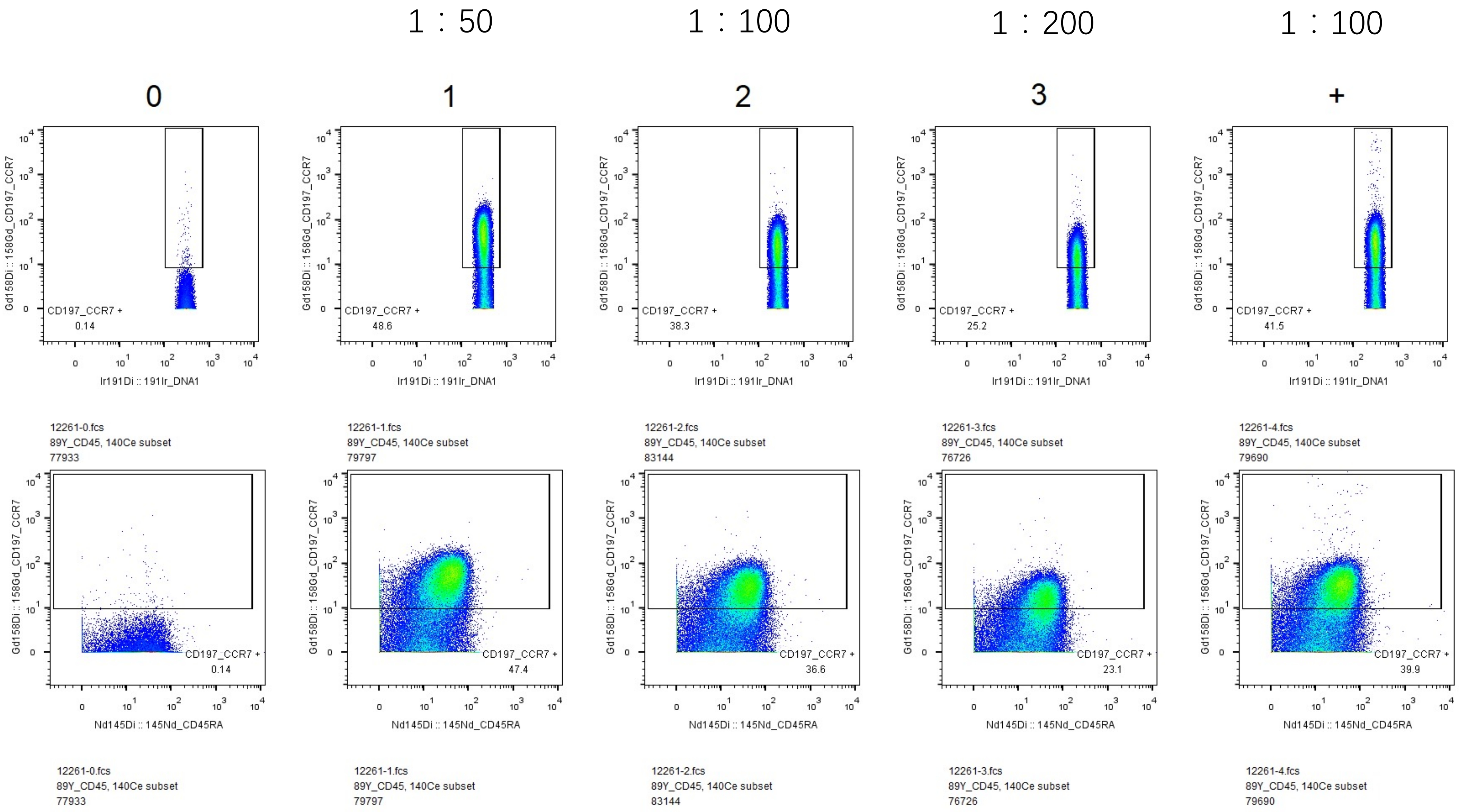

4237-159-CD11c

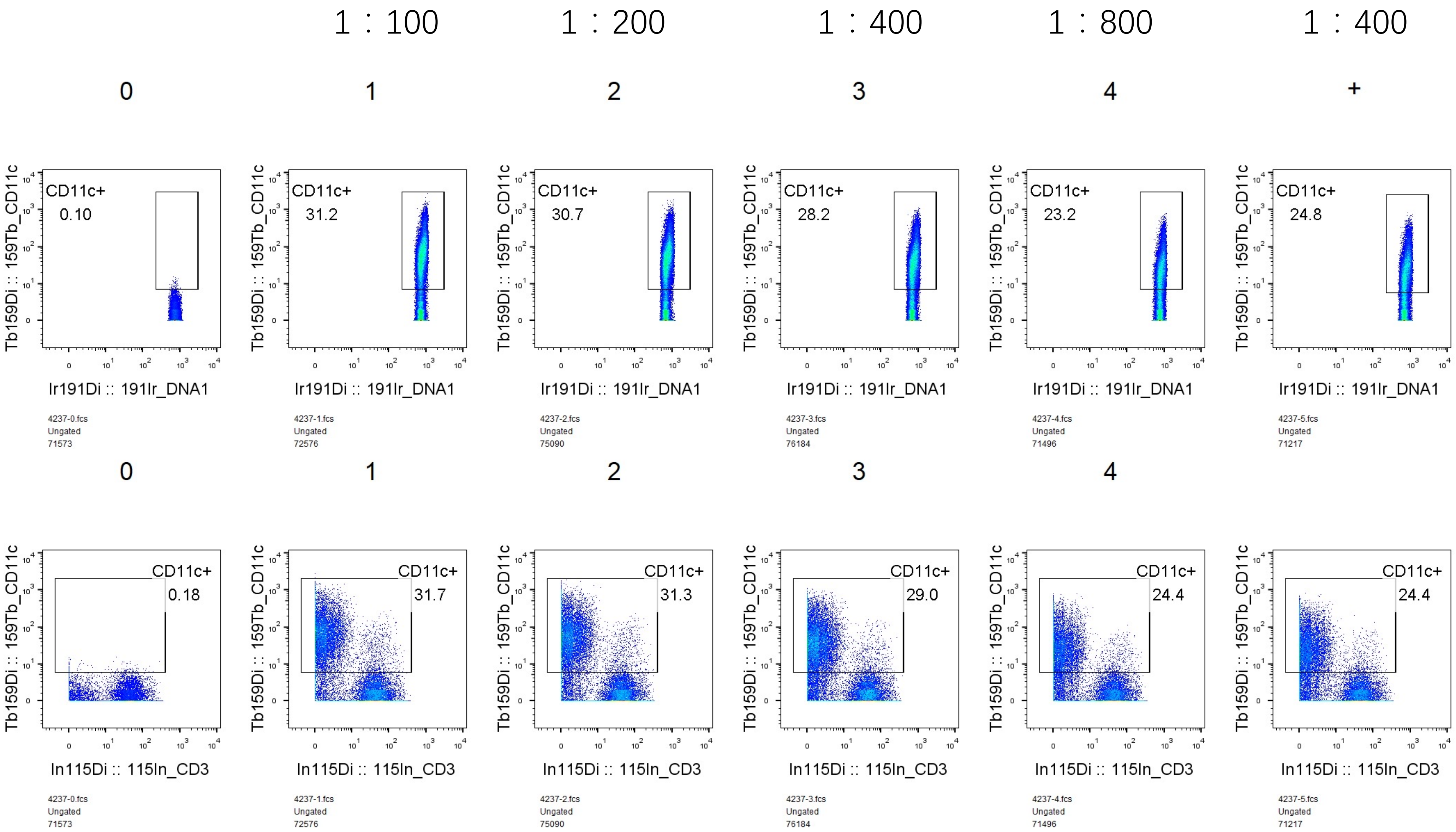

11606-160-CD33

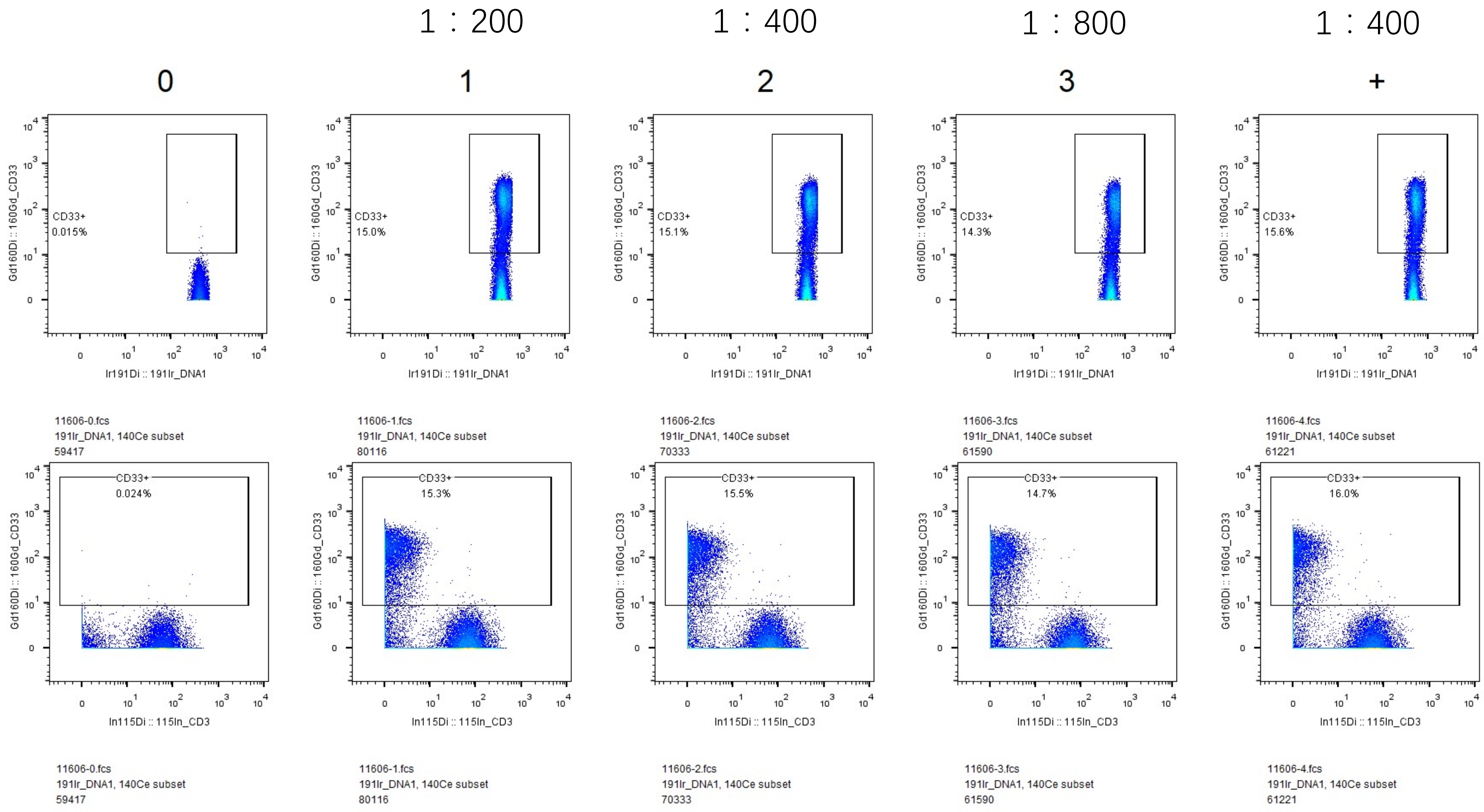

13007-161-CD152-CTLA-4

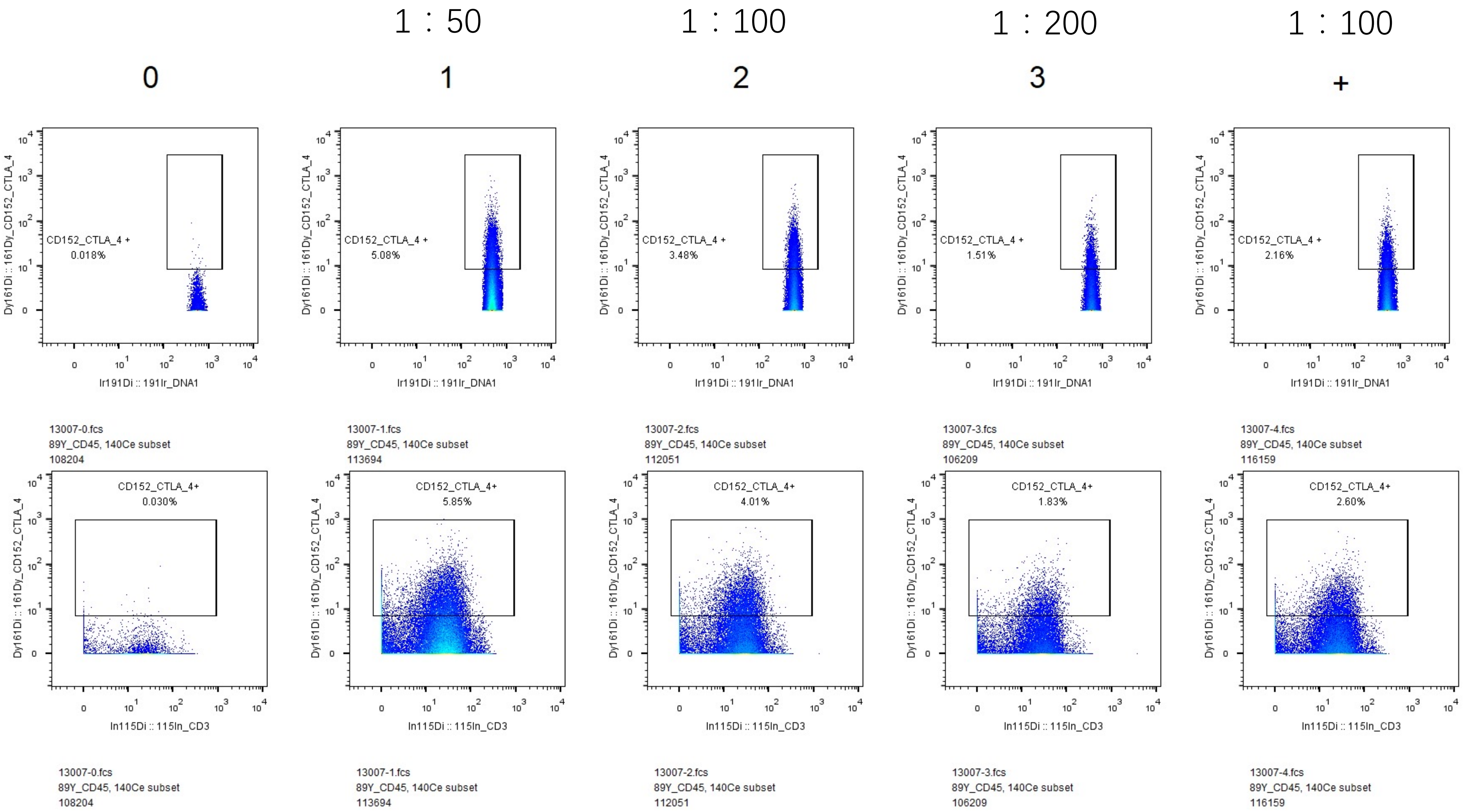

11373-162-FOXP3

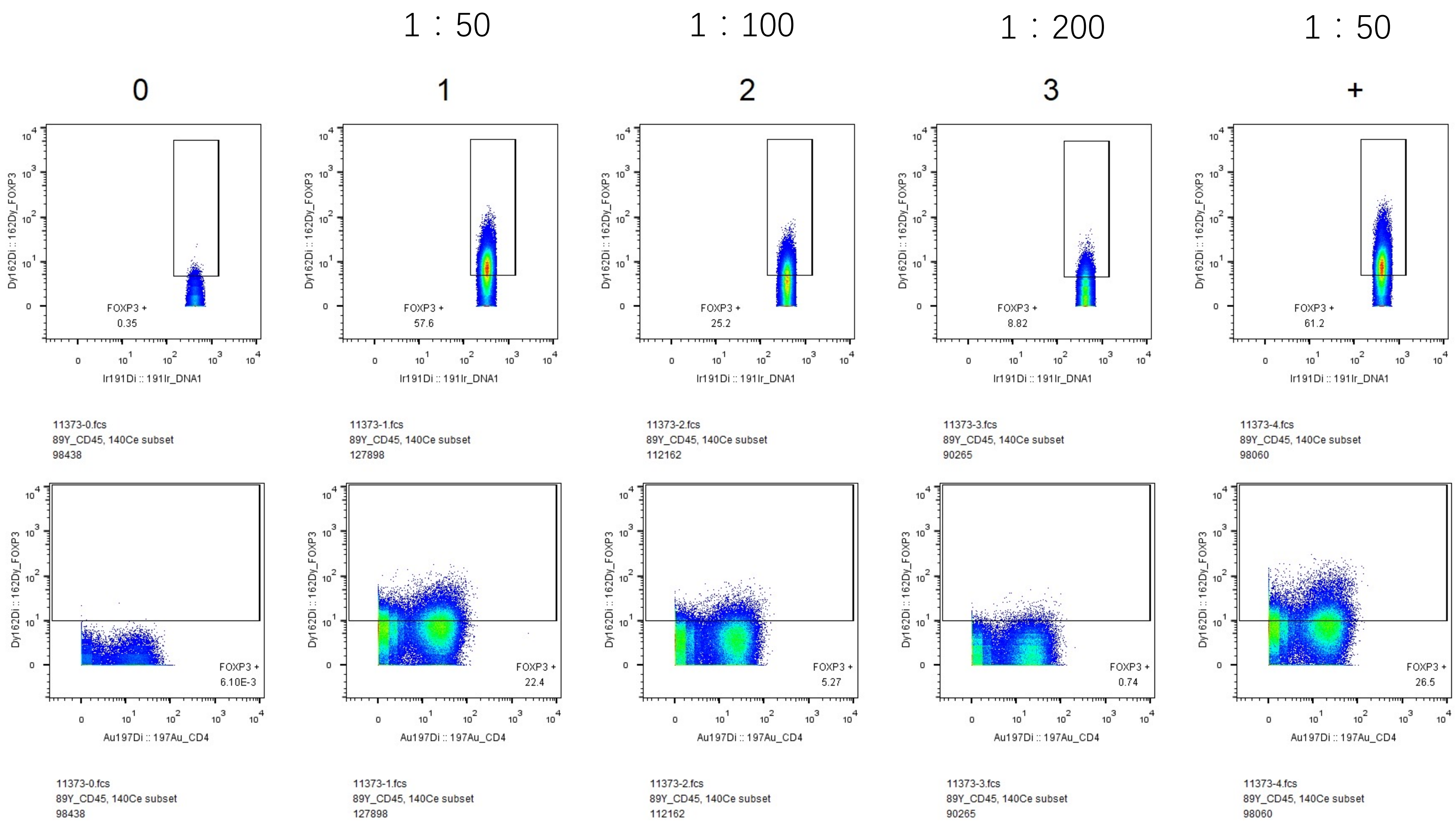

8227-163-CD183-CXCR3

0	1	2	3	+
	1 ：50	1 ：100	1 ：200	1 ：50

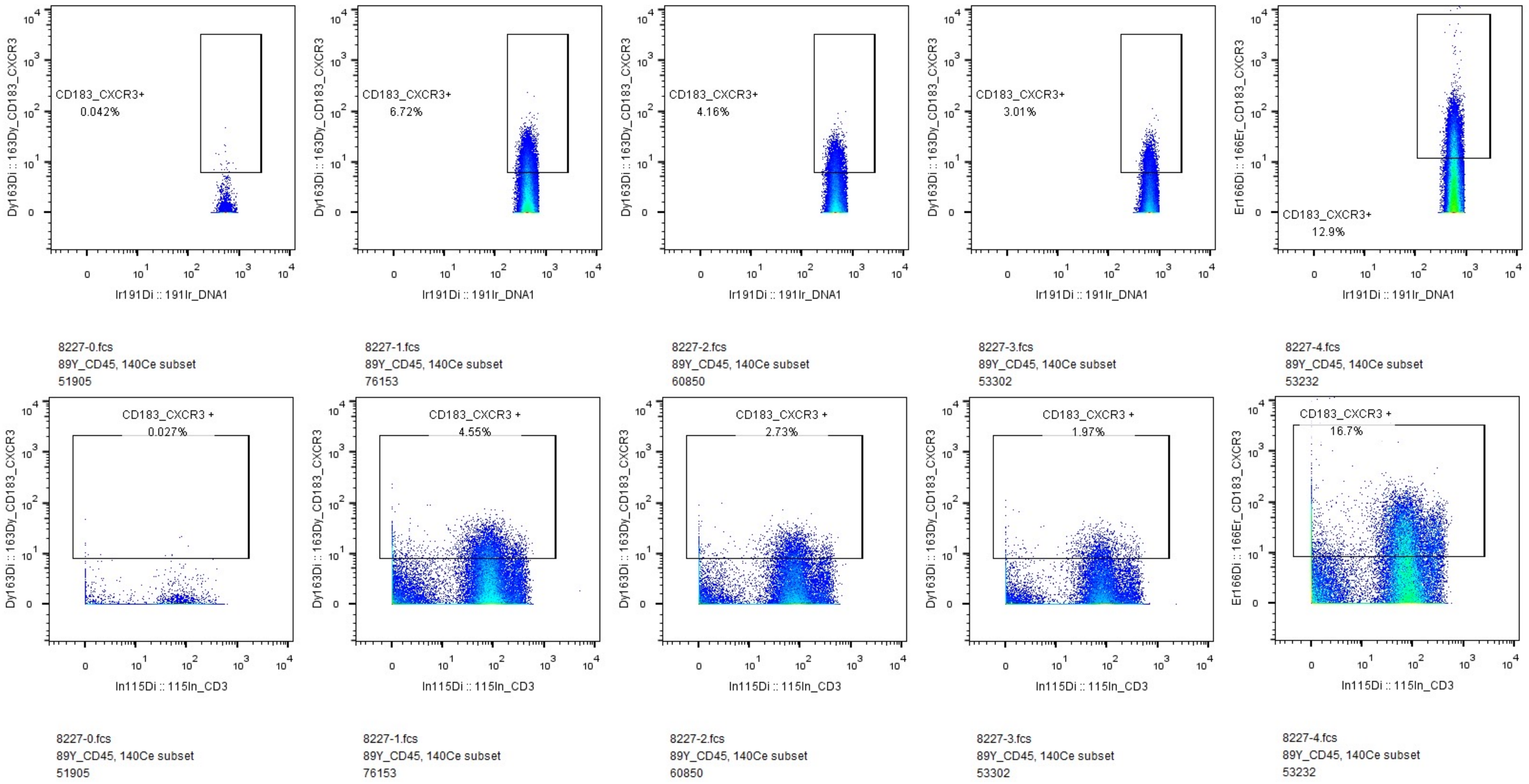

11279-164-CD185-CXCR5

1：50　　1：100　　1：200　　1：400

0　　1　　2　　3　　+

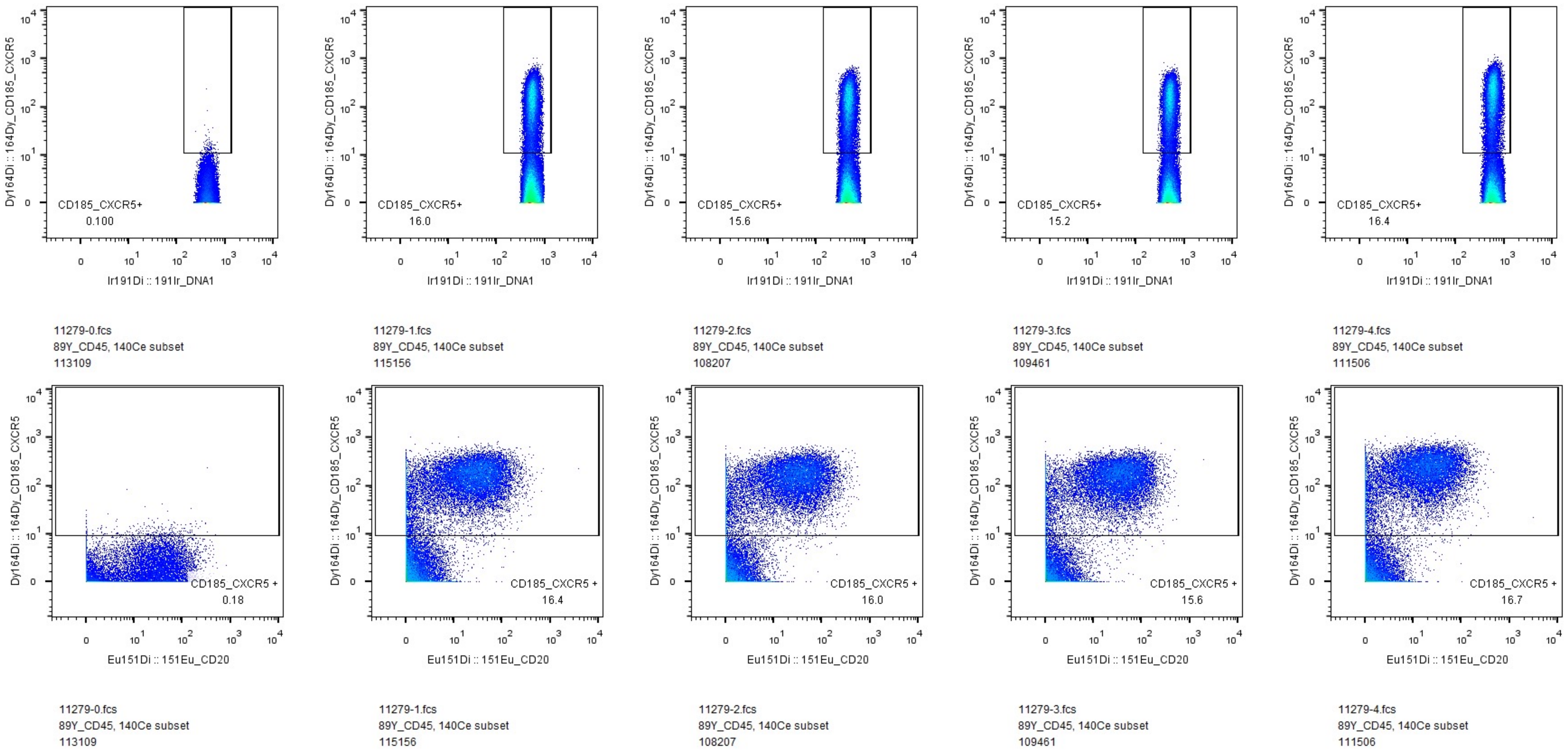

4261-165-CD66b-200311

	1：100	1：200	1：400	1：400
0	1	2	3	+

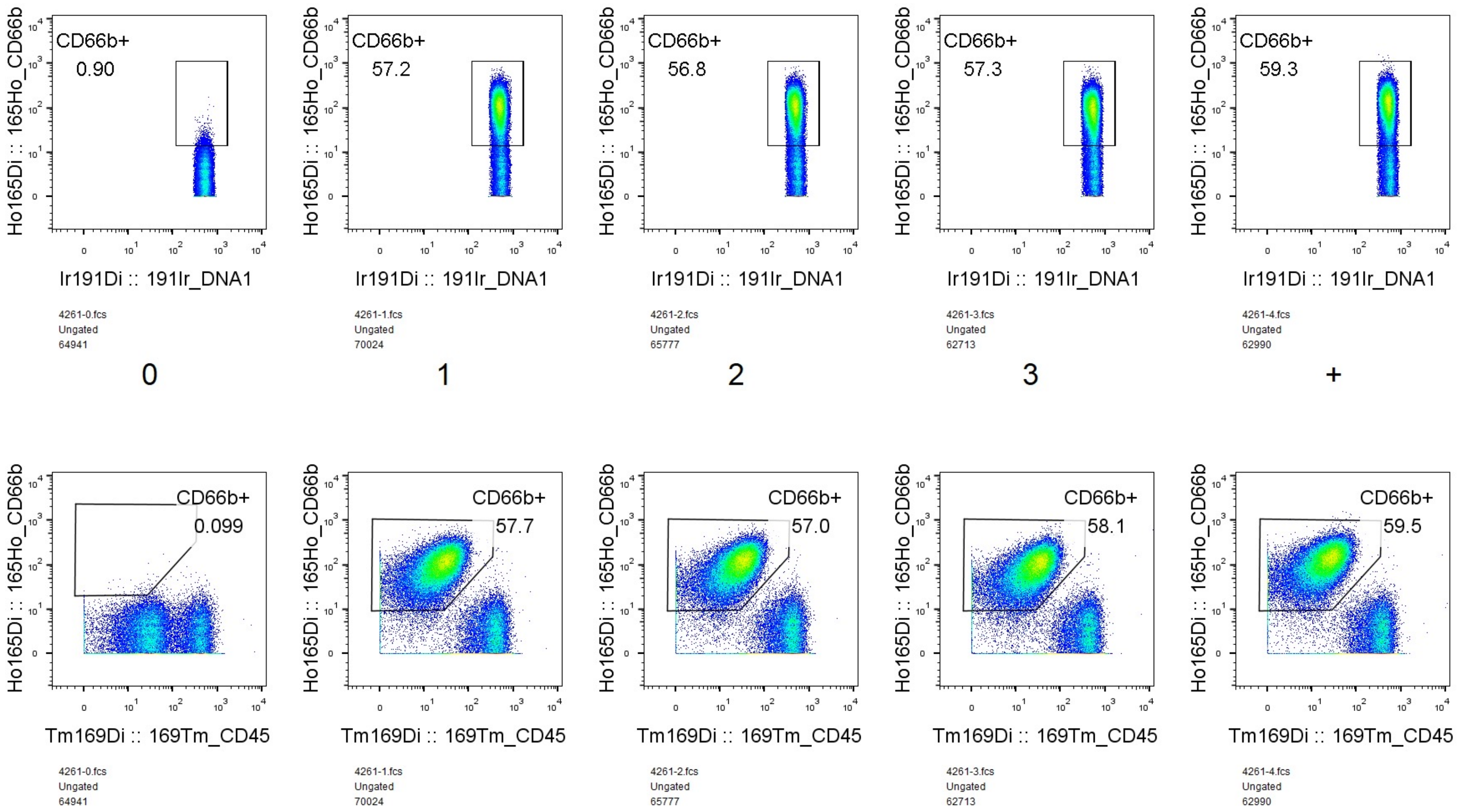

7225-166-CD134-OX40

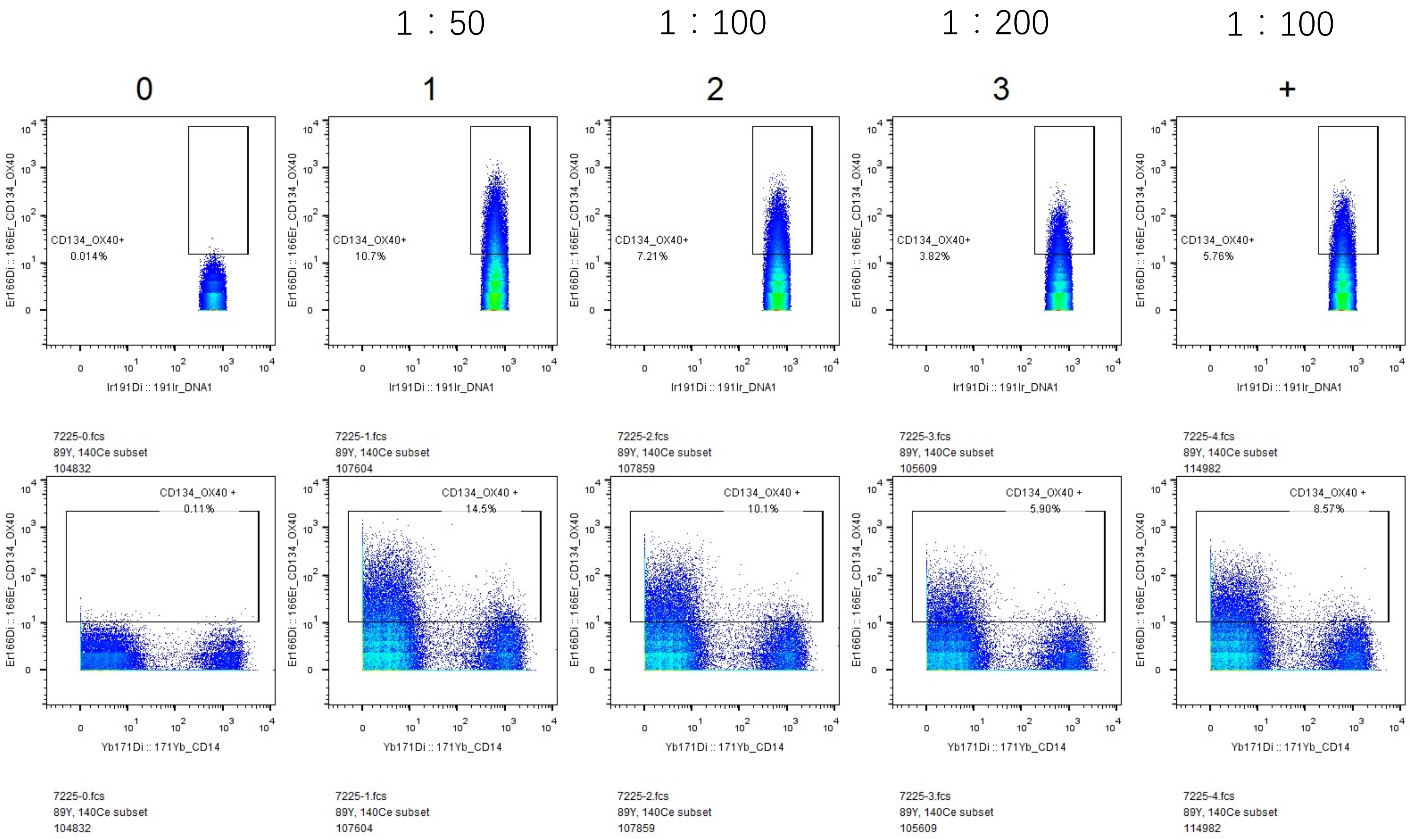

8393-167-CD27

	1 : 100	1 : 200	1 : 400	1 : 200
0	1	2	3	+

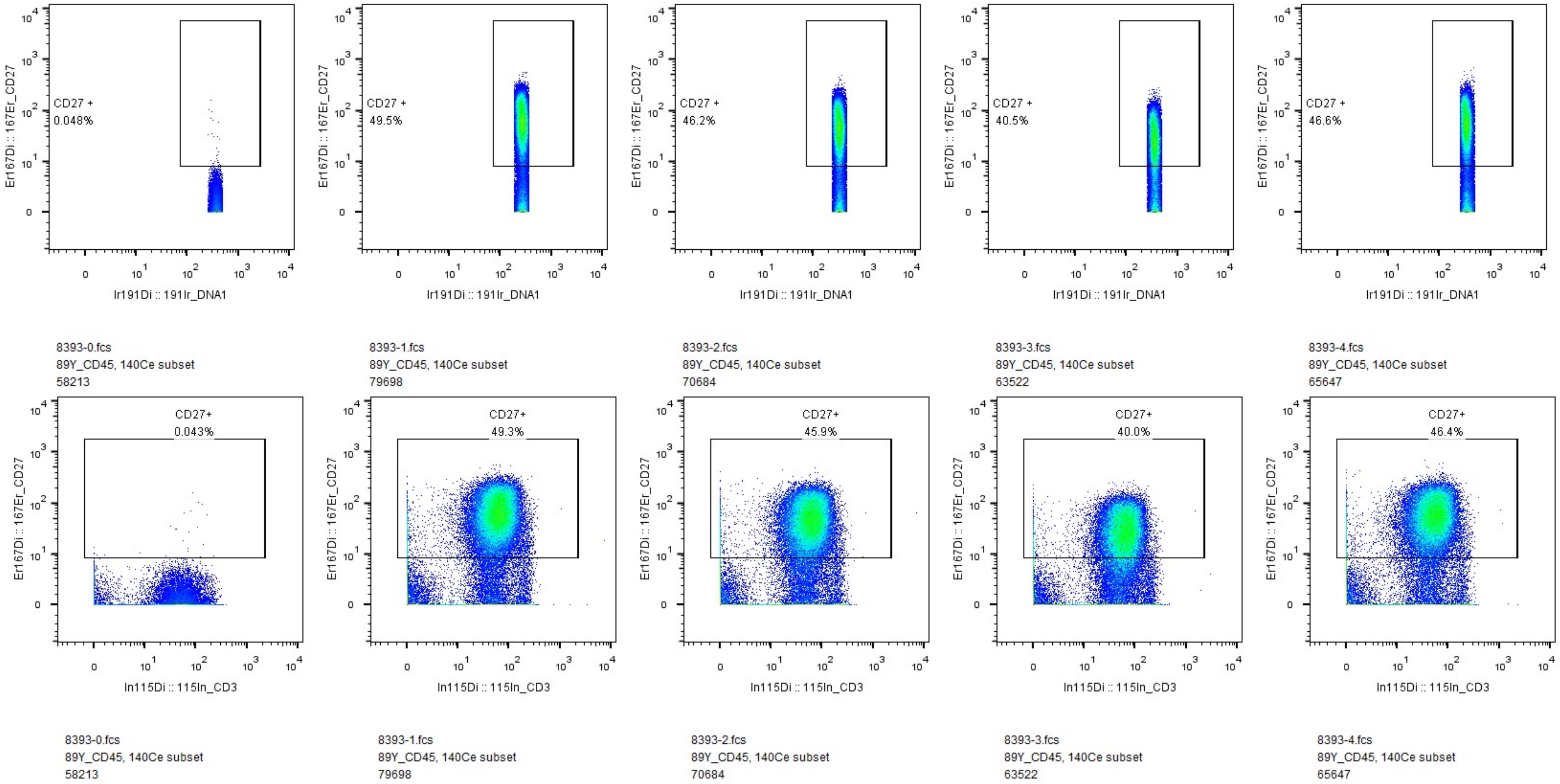

14554-168-CD69

	1 : 50	1 : 100	1 : 200	1 : 200
0	1	2	3	+

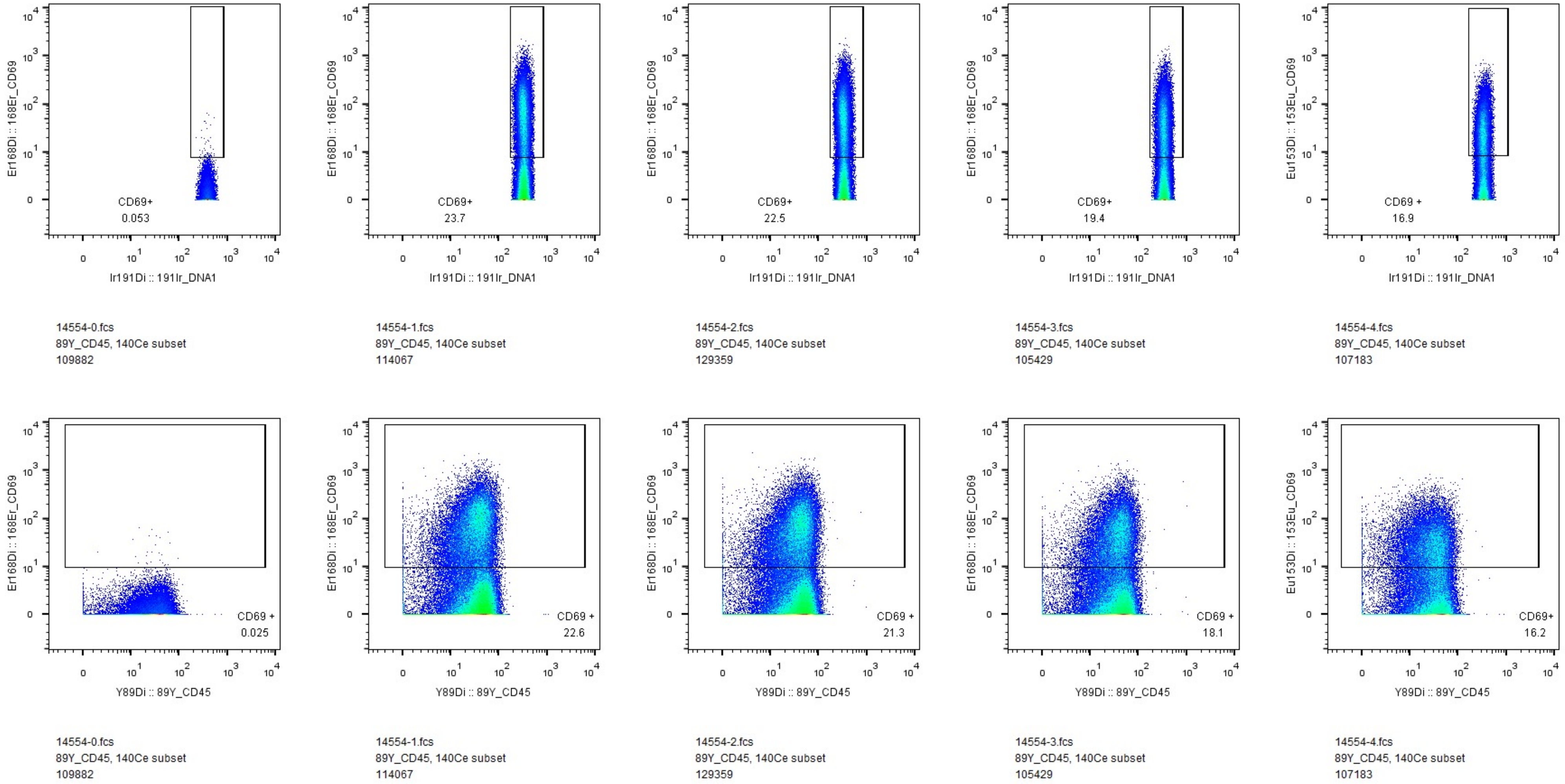

11279-169-Ki-67

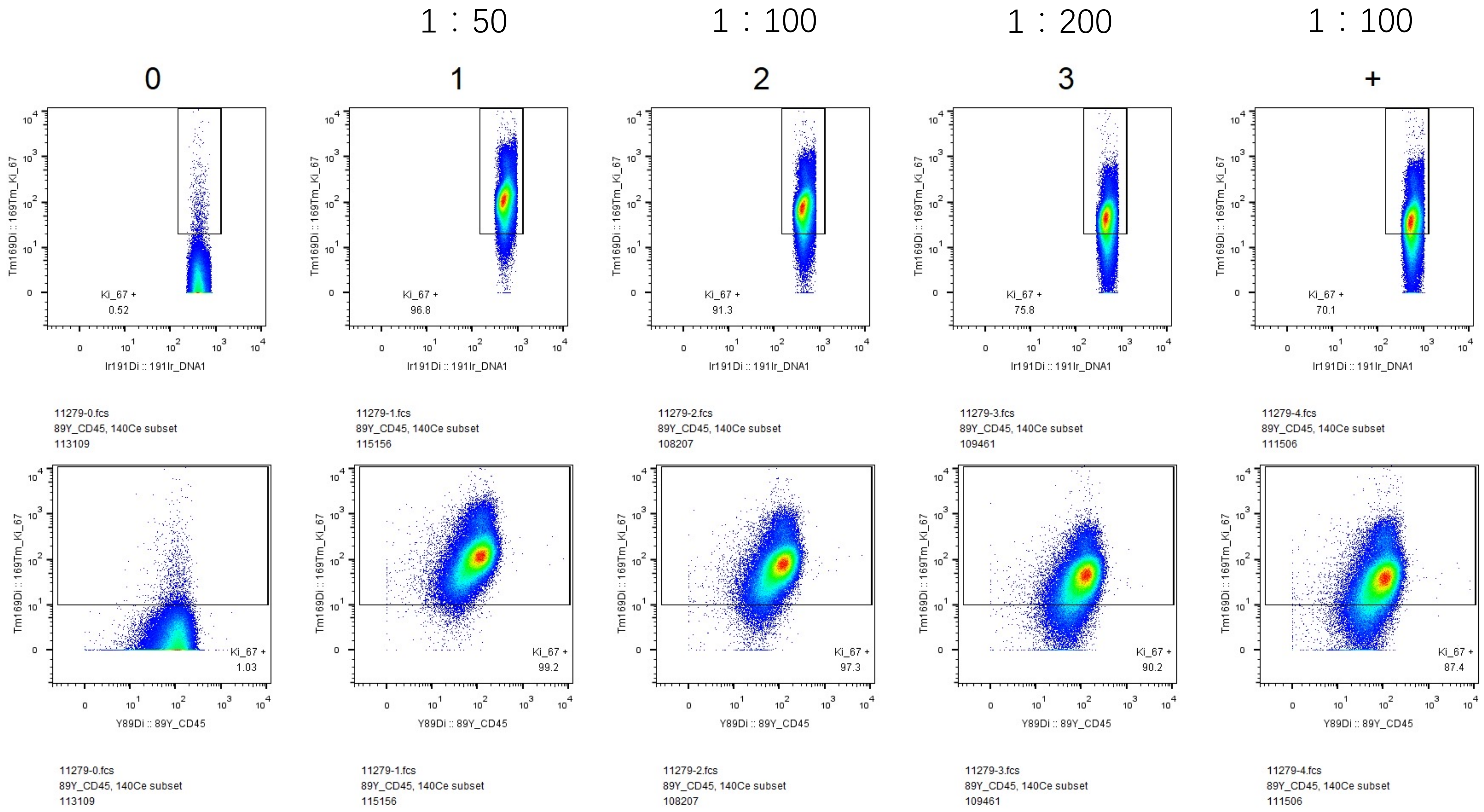

10638-170-T-bet

	1：100	1：200	1：400	1：200
0	1	2	3	+

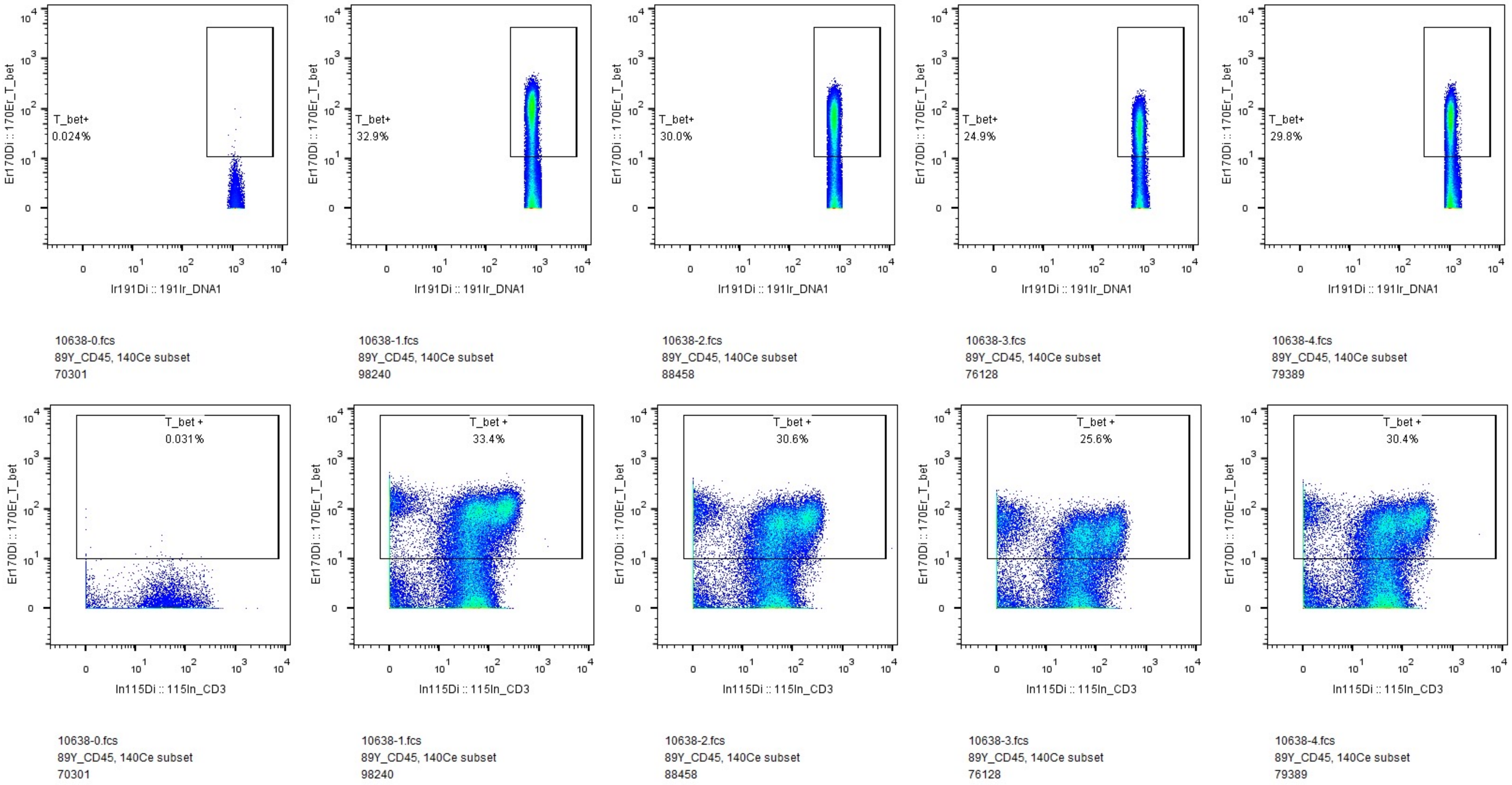

12379-171-EOMES

	1：50	1：100	1：200	1：50
0	1	2	3	+

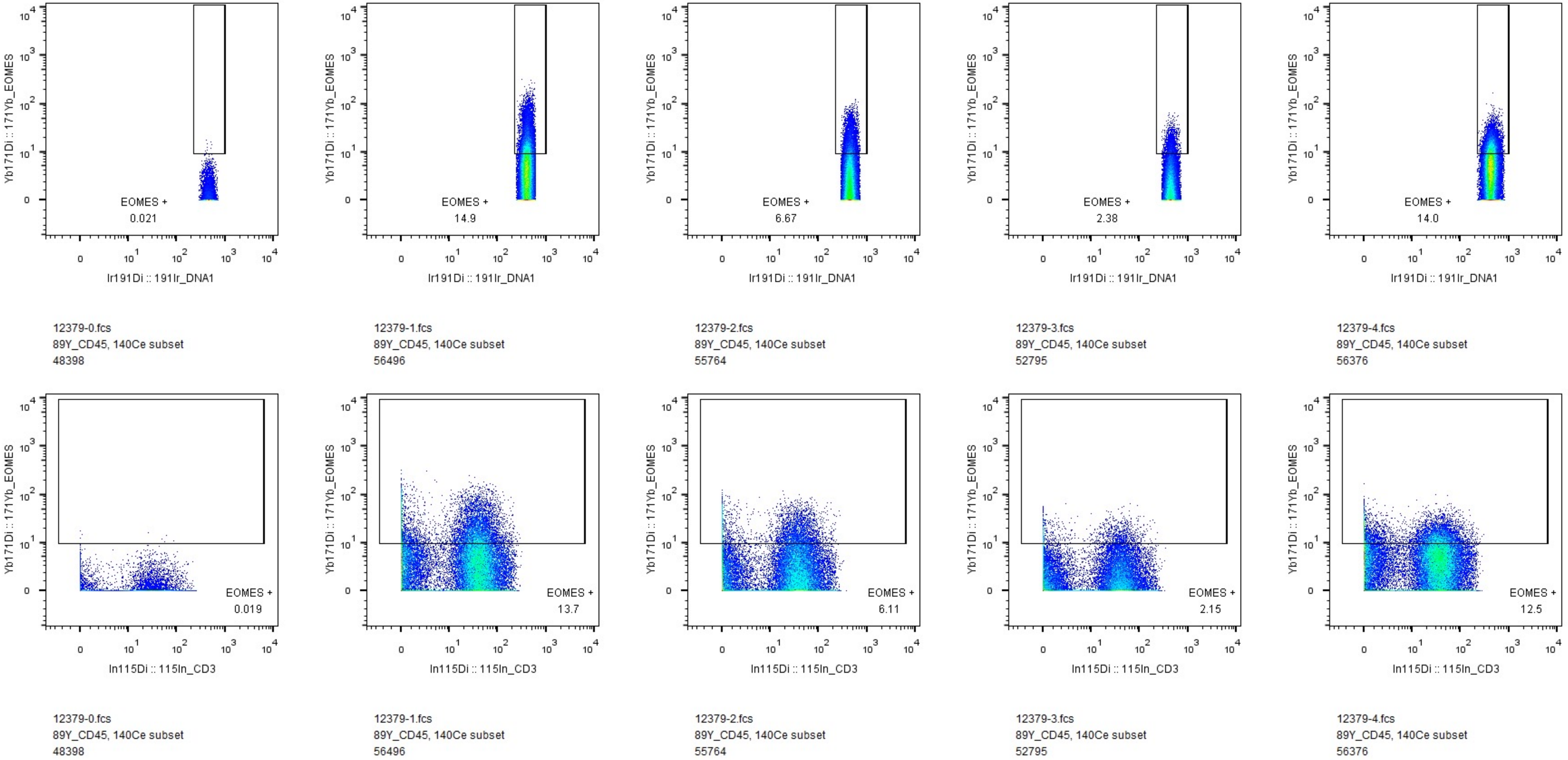

13358-172-CD38

	1：50	1：100	1：200	1：50
**0**	**1**	**2**	**3**	**+**

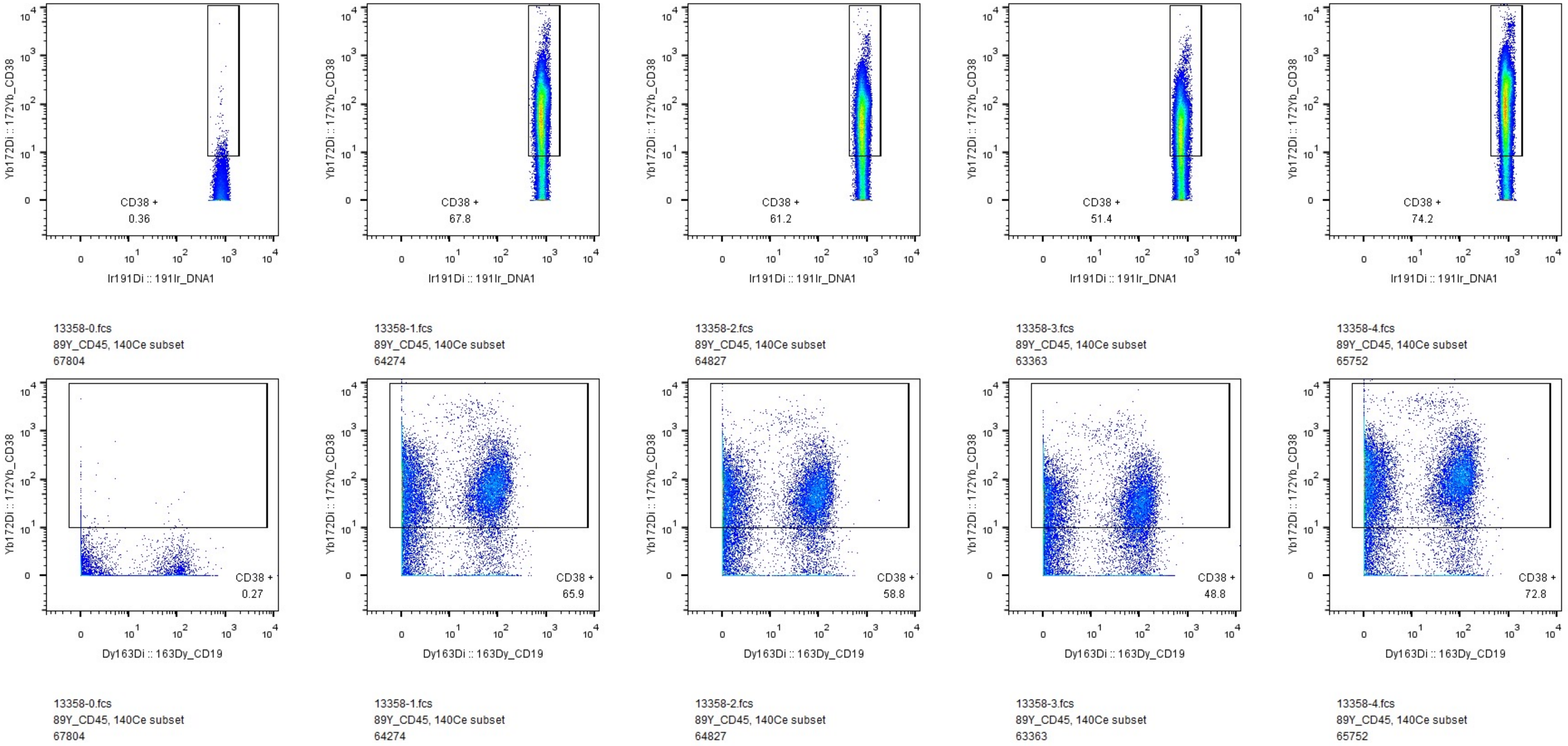

11845-173-GranzymeB

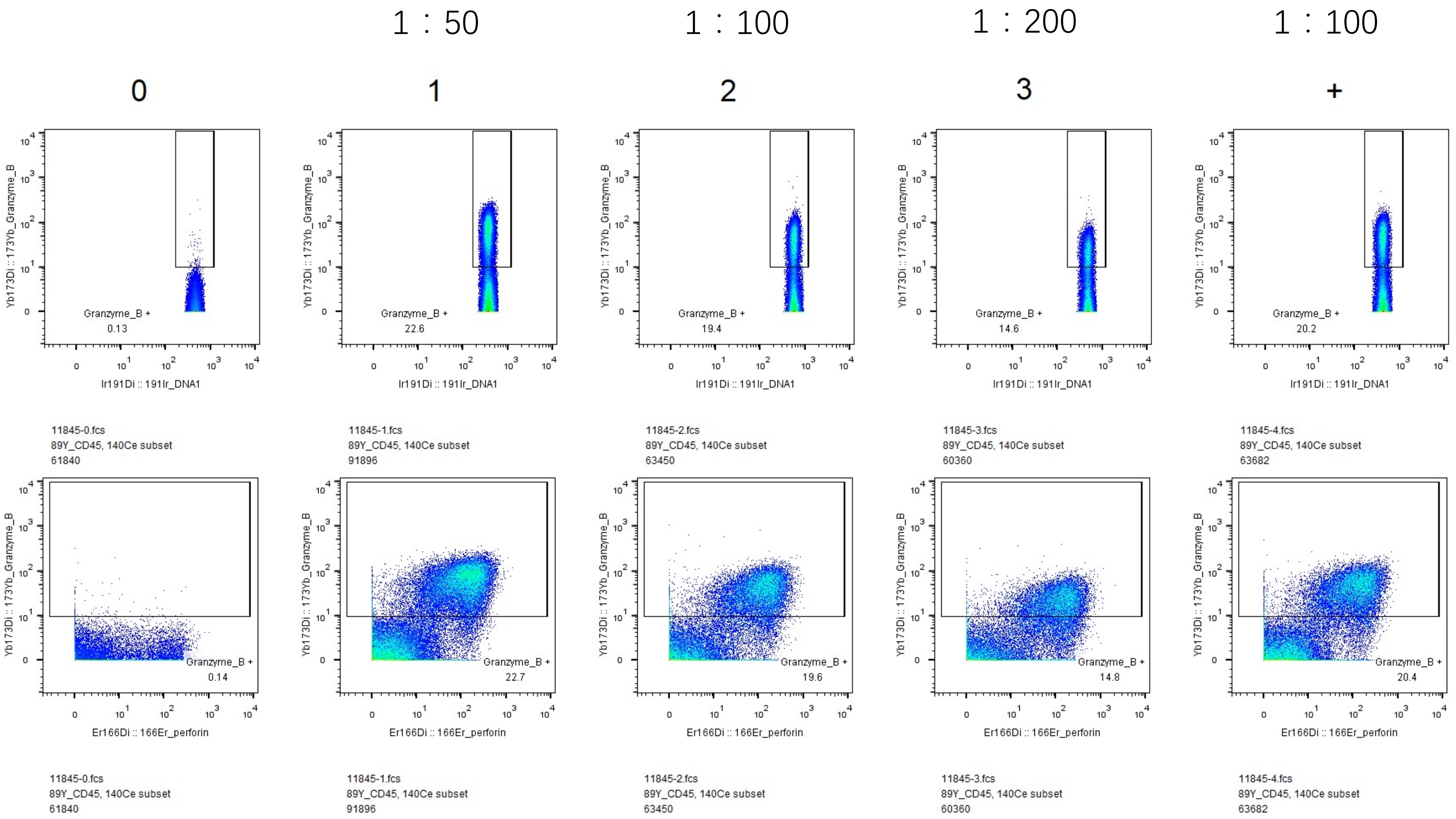

11670-174-CD279-PD-1

0	1 (1：50)	2 (1：100)	3 (1：200)	+ (1：50)

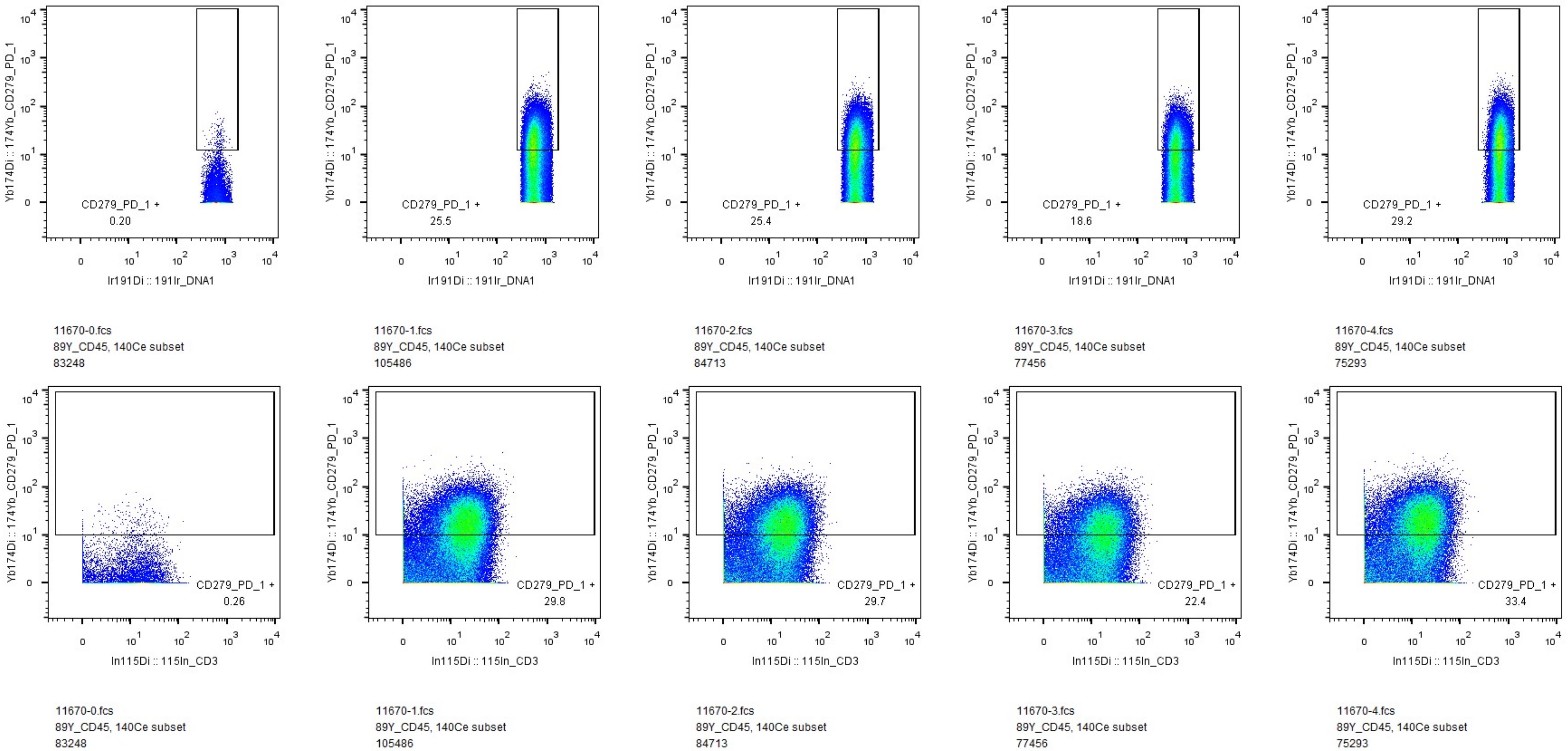

13886-175-CD16

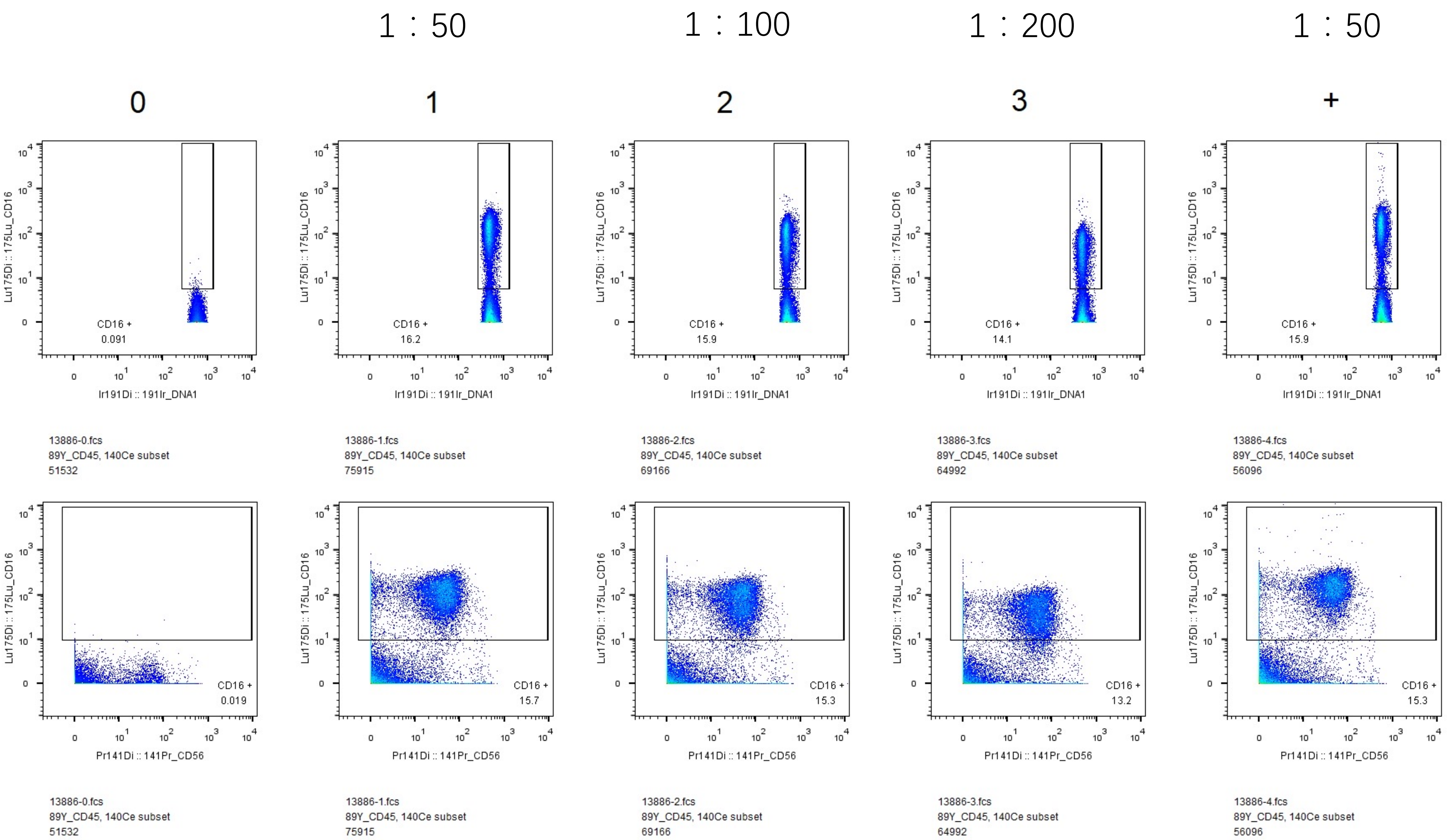

13094-176-HLA-DR

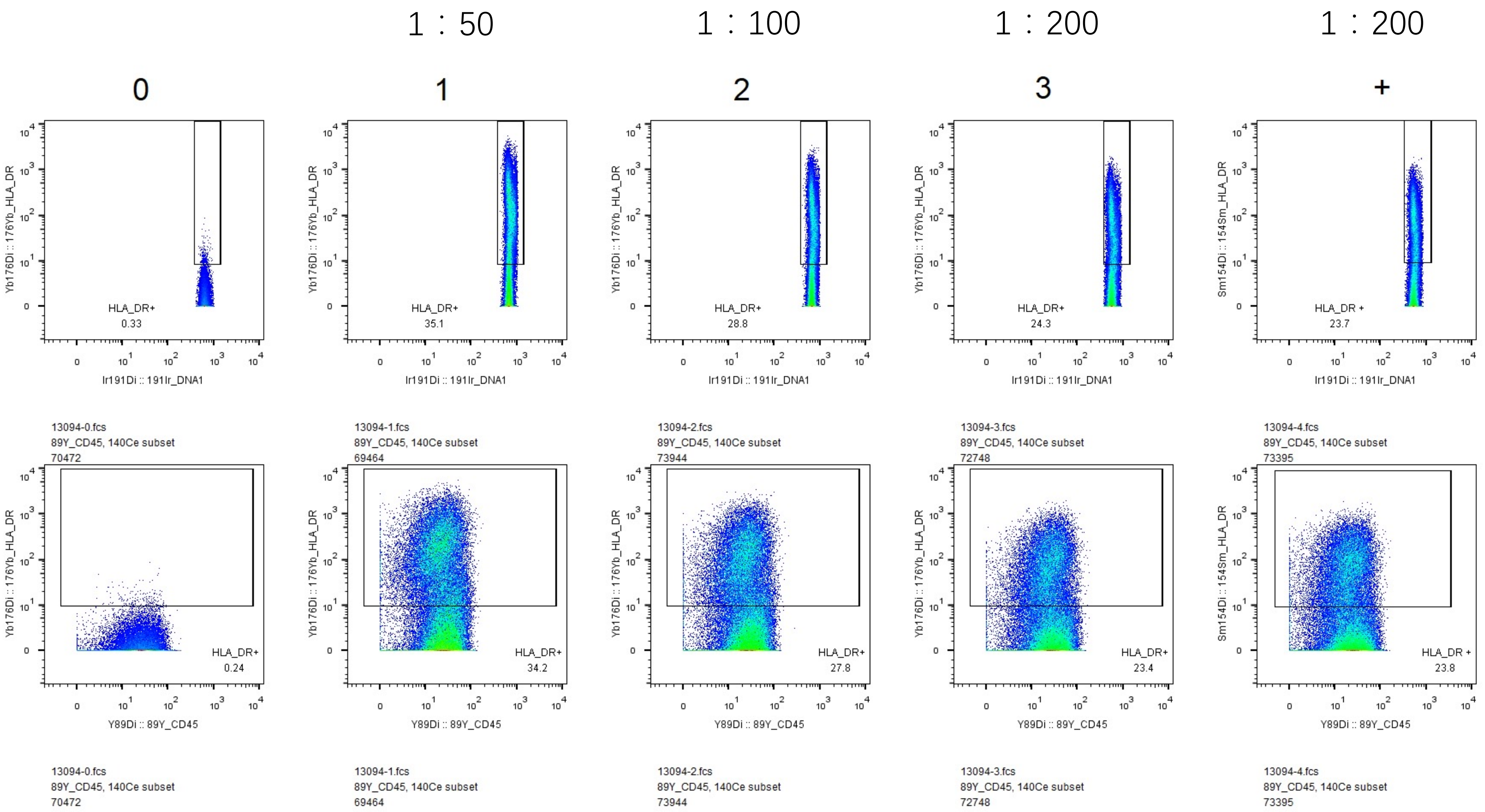

14000-197-CD4

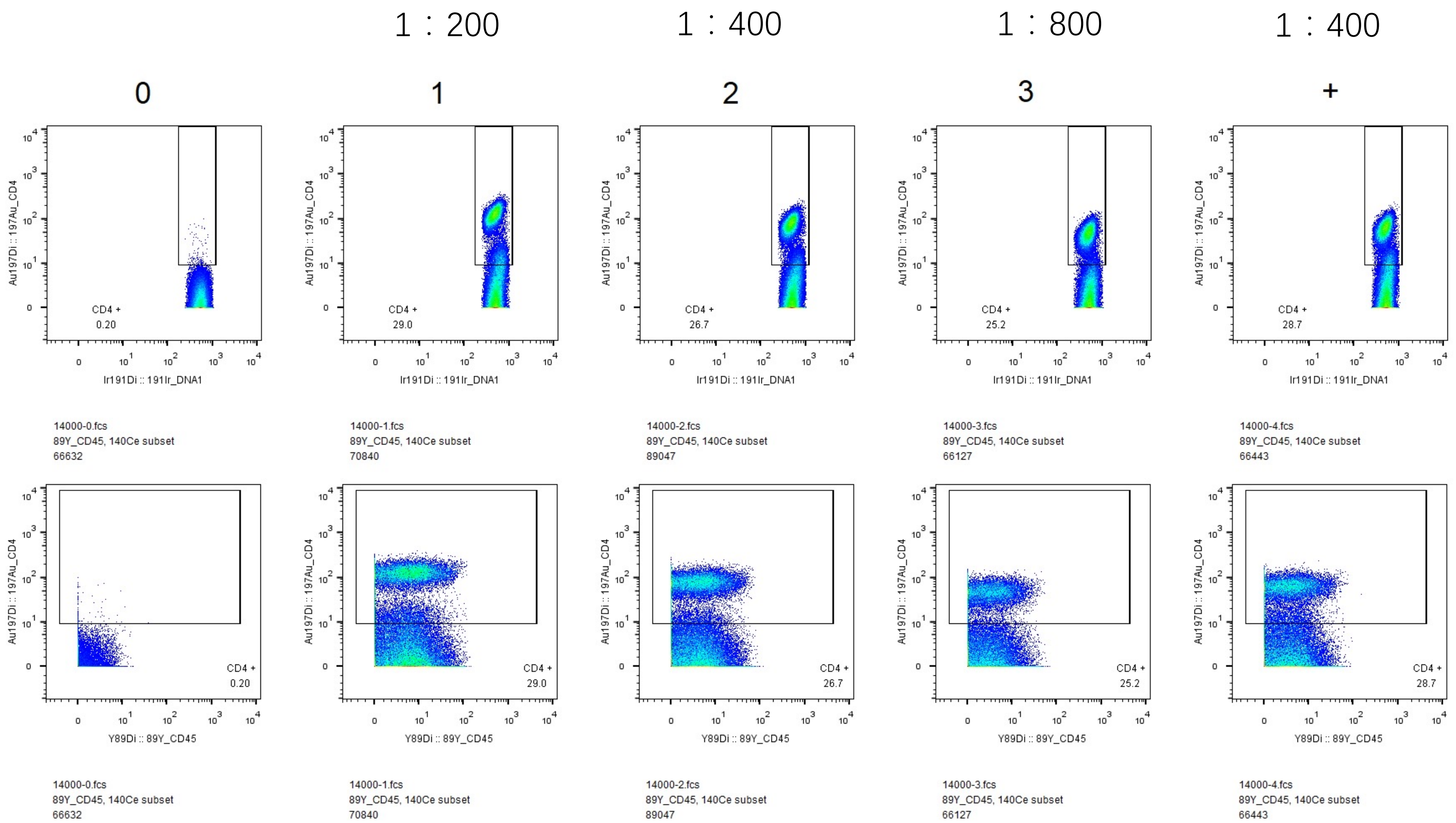

14222-198-CD8

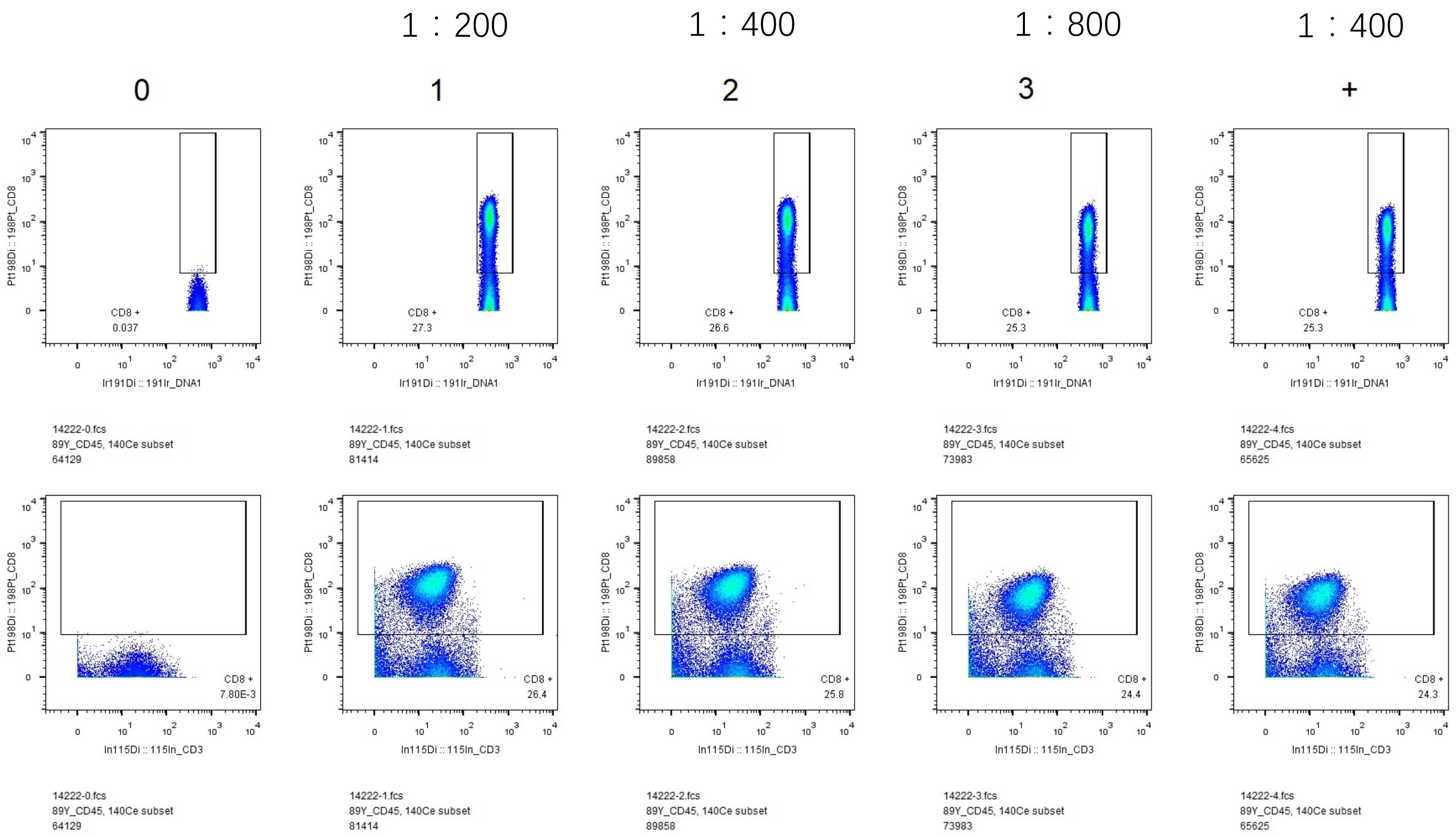

14222-209-CD11b

0	1 : 200	1 : 400	1 : 800	1 : 400
0	1	2	3	+

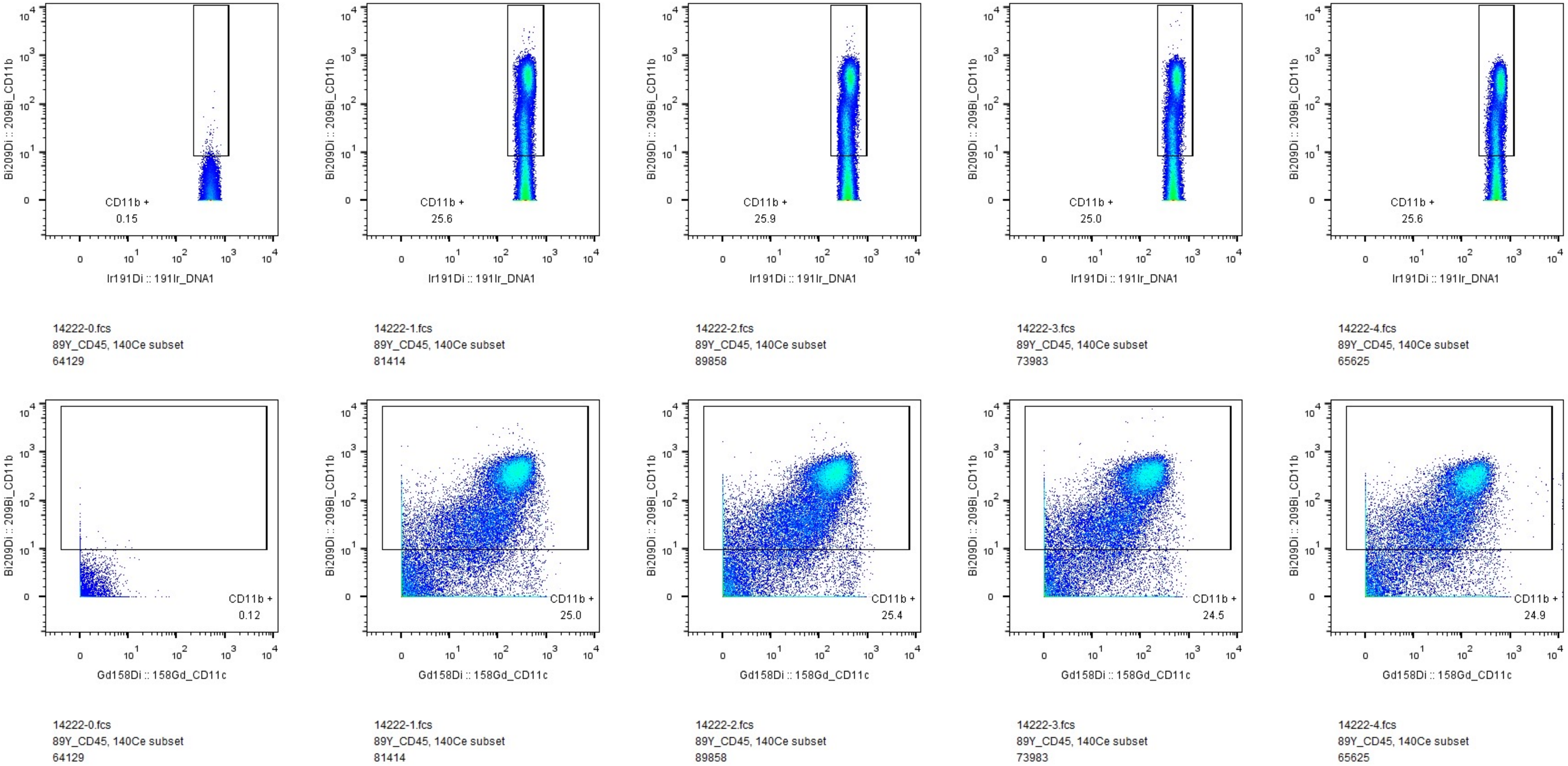

Supplement: Supplementary file 1 [file Image_1.pdf]
