# Supplementary figures and images for: Phenotypic and functional alteration of CD45+ immune cells in the decidua of preeclampsia patients analyzed by mass cytometry (CyTOF)
Source: Front Immunol. 2023 Jan 6;13:1047986. doi: 10.3389/fimmu.2022.1047986 (PMC9852836; doi:10.3389/fimmu.2022.1047986)

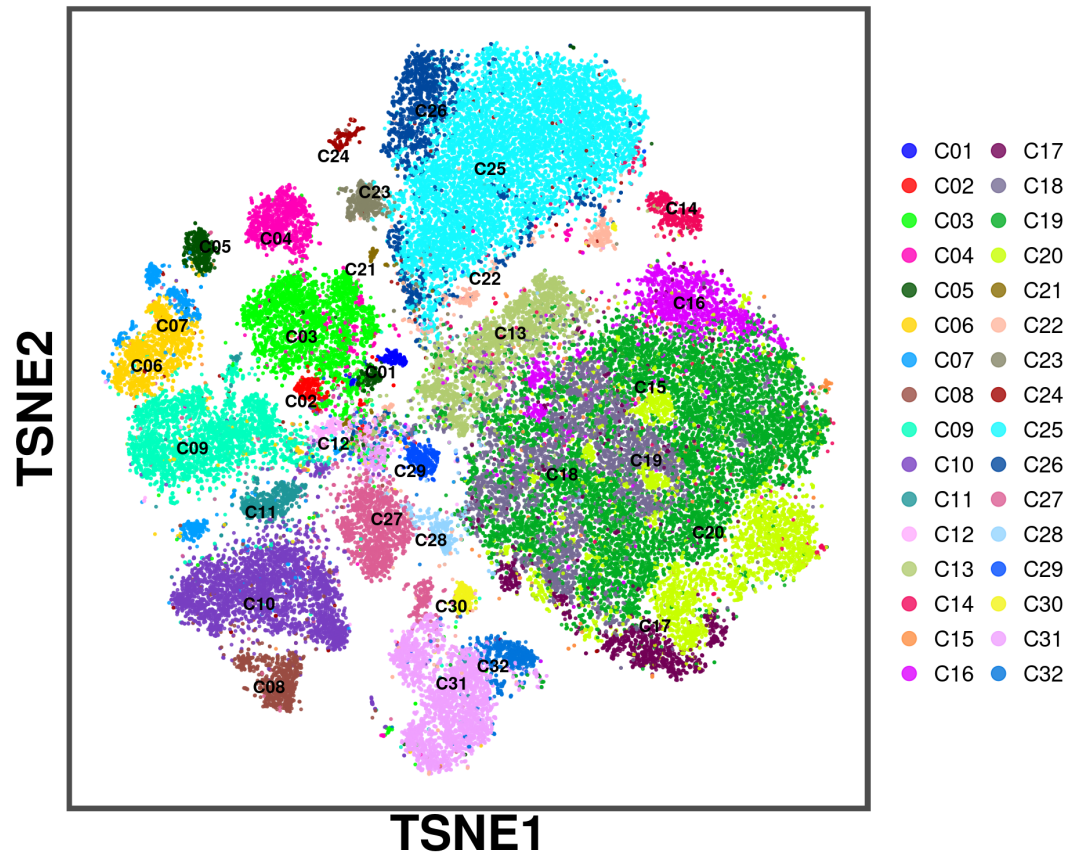

**Figure S2.** A two-dimensional t-SNE map is obtained to visualize the clustering of all immune cells.

Supplement: Supplementary file 2 [file Image_2.pdf]
