# Supplementary material for: Phenotypic and functional alteration of CD45+ immune cells in the decidua of preeclampsia patients analyzed by mass cytometry (CyTOF)
Source: Front Immunol. 2023 Jan 6;13:1047986. doi: 10.3389/fimmu.2022.1047986 (PMC9852836; doi:10.3389/fimmu.2022.1047986)
Supplement: Supplementary file 3 [file Image_3.pdf]

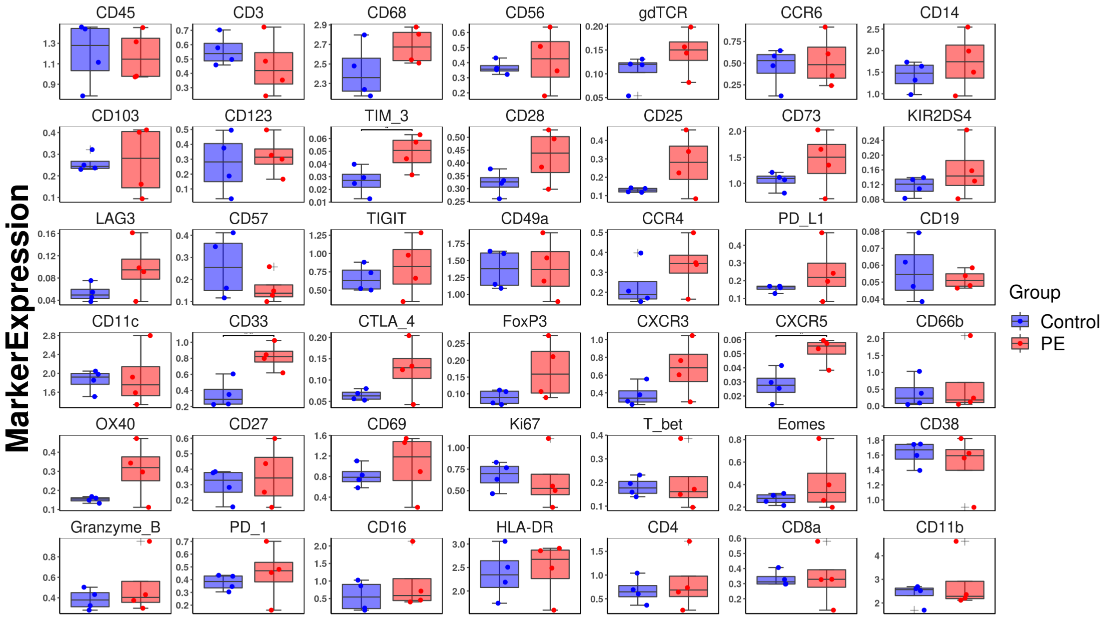

Figure S3. All 42 markers expressed in CD45+ decidual cells of PE and control groups. The blue bar and red bar denote control and PE group, respectively. * stands for p value < 0.01. ** stands for p value < 0.001.
