# Supplementary material for: Phenotypic and functional alteration of CD45+ immune cells in the decidua of preeclampsia patients analyzed by mass cytometry (CyTOF)
Source: Front Immunol. 2023 Jan 6;13:1047986. doi: 10.3389/fimmu.2022.1047986 (PMC9852836; doi:10.3389/fimmu.2022.1047986)
Supplement: Supplementary file 4 [file Image_4.pdf]

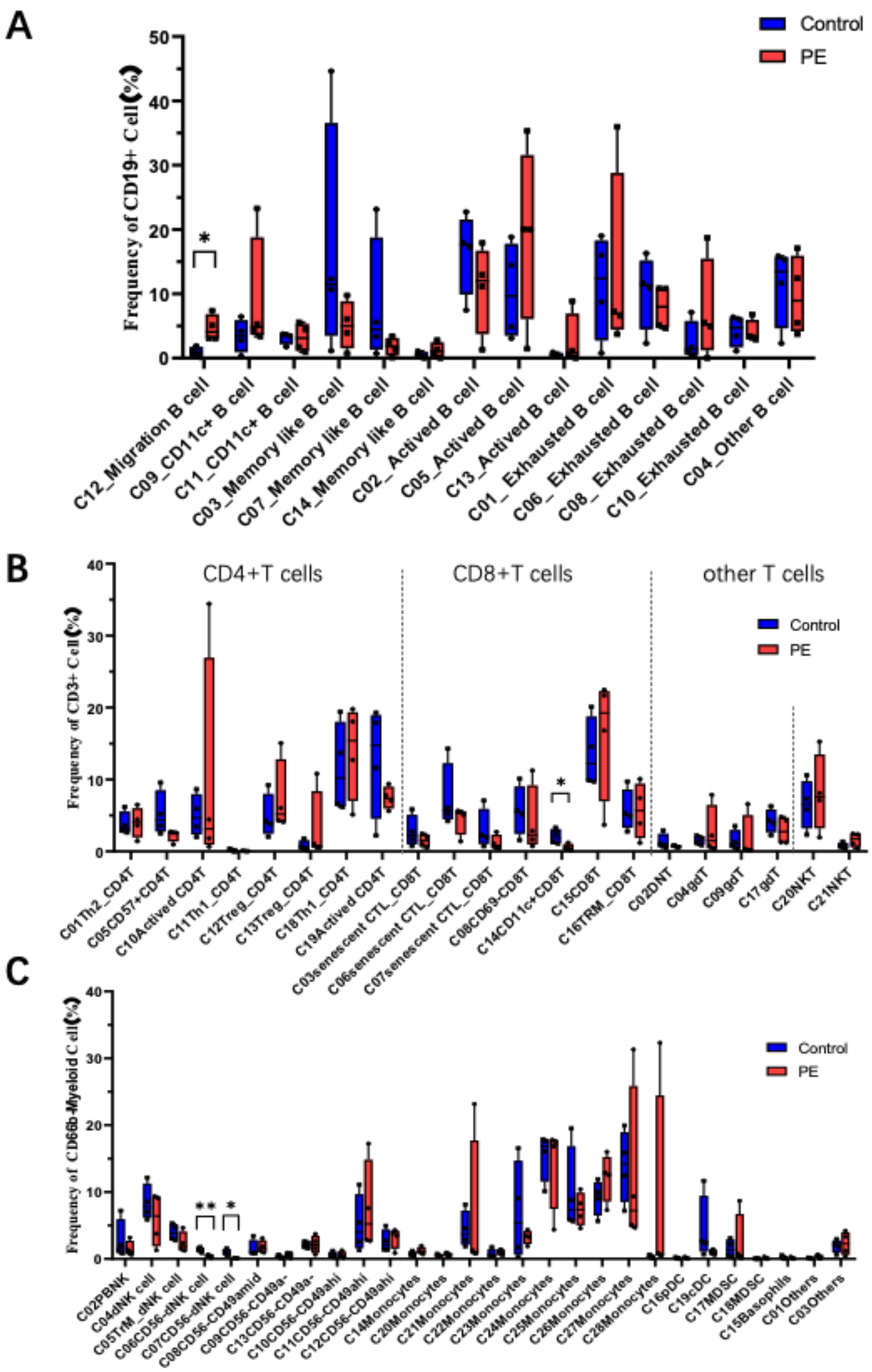

Figure S4. Bar plots of the frequencies of each cluster obtained from re-clustering of CD19+ B cells (A), CD3+ T cells (B) and CD45+CD3- CD19- CD66b- Myeloid cells. The blue bar and red bar denote control and PE group, respectively. * stands for p value < 0.01.
